# Robust SNP-based prediction of rheumatoid arthritis through machine-learning-optimized polygenic risk score

**DOI:** 10.1186/s12967-023-03939-5

**Published:** 2023-02-07

**Authors:** Ashley J. W. Lim, C. Tera Tyniana, Lee Jin Lim, Justina Wei Lynn Tan, Ee Tzun Koh, TTSH Rheumatoid Arthritis Study Group, Andrea Ee Ling Ang, Grace Yin Lai Chan, Madelynn Tsu-Li Chan, Faith Li-Ann Chia, Hiok Hee Chng, Choon Guan Chua, Hwee Siew Howe, Li Wearn Koh, Kok Ooi Kong, Weng Giap Law, Samuel Shang Ming Lee, Tsui Yee Lian, Xin Rong Lim, Jess Mung Ee Loh, Mona Manghani, Sze-Chin Tan, Claire Min-Li Teo, Bernard Yu-Hor Thong, Paula Permatasari Tjokrosaputro, Chuanhui Xu, Samuel S. Chong, Chiea Chuen Khor, Khai Pang Leong, Caroline G. Lee

**Affiliations:** 1 Department of Biochemistry, Yong Loo Lin School of Medicine, National University of Singapore, C/O MD7, Level 2, 8 Medical Drive, Singapore, 117597 Singapore; 2 Department of Bioinformatics, School of Life Sciences, Indonesia International Institute for Life Sciences, Jakarta, Indonesia; 3 Department of Rheumatology, Allergy and Immunology, Tan Tock Seng Hospital, Singapore, Singapore; 4 Dept of Pediatrics and Obstetrics & Gynecology, Yong Loo Lin School of Medicine, National University of Singapore, Singapore, Singapore; 5 Division of Human Genetics, Genome Institute of Singapore, Singapore, Singapore; 6 Div of Cellular & Molecular Research, Humphrey Oei Institute of Cancer Research, National Cancer Centre Singapore, Singapore, Singapore; 7 Duke-NUS Medical School, Singapore, Singapore; 8 NUS Graduate School, National University of Singapore, Singapore, Singapore; 9 Clinical Research & Innovation Office, Tan Tock Seng Hospital, Singapore, Singapore

**Keywords:** Machine-learning, Polygenic risk score, Rheumatoid arthritis, Single nucleotide polymorphisms

## Abstract

**Background:**

The popular statistics-based Genome-wide association studies (GWAS) have provided deep insights into the field of complex disorder genetics. However, its clinical applicability to predict disease/trait outcomes remains unclear as statistical models are not designed to make predictions. This study employs statistics-free machine-learning (ML)-optimized polygenic risk score (PRS) to complement existing GWAS and bring the prediction of disease/trait outcomes closer to clinical application. Rheumatoid Arthritis (RA) was selected as a model disease to demonstrate the robustness of ML in disease prediction as RA is a prevalent chronic inflammatory joint disease with high mortality rates, affecting adults at the economic prime. Early identification of at-risk individuals may facilitate measures to mitigate the effects of the disease.

**Methods:**

This study employs a robust ML feature selection algorithm to identify single nucleotide polymorphisms (SNPs) that can predict RA from a set of training data comprising RA patients and population control samples. Thereafter, selected SNPs were evaluated for their predictive performances across 3 independent, unseen test datasets. The selected SNPs were subsequently used to generate PRS which was also evaluated for its predictive capacity as a sole feature.

**Results:**

Through robust ML feature selection, 9 SNPs were found to be the minimum number of features for excellent predictive performance (AUC > 0.9) in 3 independent, unseen test datasets. PRS based on these 9 SNPs was significantly associated with (P < 1 × 10^–16^) and predictive (AUC > 0.9) of RA in the 3 unseen datasets. A RA ML-PRS calculator of these 9 SNPs was developed (https://xistance.shinyapps.io/prs-ra/) to facilitate individualized clinical applicability. The majority of the predictive SNPs are protective, reside in non-coding regions, and are either predicted to be potentially functional SNPs (pfSNPs) or in high linkage disequilibrium (r2 > 0.8) with un-interrogated pfSNPs.

**Conclusions:**

These findings highlight the promise of this ML strategy to identify useful genetic features that can robustly predict disease and amenable to translation for clinical application.

**Supplementary Information:**

The online version contains supplementary material available at 10.1186/s12967-023-03939-5.

## Introduction

Over the past few decades, genome wide association studies (GWAS) have revolutionised the field of complex disorder genetics, with the identification of more than 70,000 significant association (p ≤ 5 × 10^–8^) of variants with diverse diseases and traits (GWAS catalogue as of April 2022) [1]. While providing deep insight into complex diseases, significant challenges remain before GWAS findings can be clinically applicable. Current GWAS employs statistical approaches to identify variants associated with a phenotype [2], based on population inferred relationship between data and the outcome variable [3]. While statistical models are able to make predictions, predictive accuracy is neither their aim nor their strength [4]. As such, clinical applicability of a disease-associated variant is less clear since statistically significant association identified in one set of data may not necessarily apply in a future dataset [5–7]. Classical statistics was designed for the analyses of data with moderate number of dependent and independent variables [3]. However, GWAS interrogates hundreds of thousands to millions of SNPs (single nucleotide polymorphisms) for disease association in an often limited number of samples. This analysis is prone to type-I errors and provides imprecise statistical inferences about the complex associations and relationships among the many variables [3]. Two strategies are used in GWAS to reduce the multiple testing and the subsequent type-I error burden. To reduce the number of variables being examined, current GWAS focuses on interrogating mainly tag-SNPs, removing other SNPs that are in strong linkage disequilibrium (LD) [8]. The second strategy to address type-I error is to employ Raw P-value Thresholding (RPVT) [9], where statistical significance of association of each SNP to phenotype is evaluated through a predefined threshold after multiple test correction. The criticisms of GWAS are that it may detect association that is spurious [10, 11], may not identify the causal variant/gene [10] and only accounts for a small fraction of the heritability of complex traits [8, 12]. These problems could be due to the removal of variants from analysis to mitigate the multiple-testing burden, as well as the treatment of variants as individual and independent, without consideration for potential higher order interactions amongst them [13, 14]. To address these criticisms, there are recent attempts to combine variants identified in GWAS to estimate the genetic risk for a trait using polygenic risk score (PRS) [15], which employs a fixed model that sums the contribution of a group of risk alleles for a specific complex disorder [16, 17], either as weighted PRS based on (p-value) and/or effect size (odds ratio) or unweighted PRS [16]. Initial attempts which employed weighted PRS achieved only limited predictive performance [16]. While relatively easy to implement and interpret, traditional PRS is based on independent, linear combination of risk alleles and assumes normal distribution of underlying data. Hence, it may not capture non-linearity or complex interactions amongst the risk alleles [17].

In contrast, machine learning (ML) is a statistics-free approach which instead focuses on the use of algorithms to identify patterns in rich and unwieldy data [3]. While statistical models often require assumptions to be made regarding the distribution of the population or the data, ML requires minimal assumptions and is effective even in the presence of complicated nonlinear interactions [3]. ML is also effective in analysing large, complex datasets with high dimensionality, which is a challenge for traditional statistical modelling methods, as in GWAS. To address the ‘curse of dimensionality’ [18–20] in ML, feature selection can be implemented to identify a subset of features that contribute most to the prediction of a variable, restricting the overall dimensionality of the dataset to only features (SNPs) that are most relevant to the prediction variable [21]. While computationally intensive, feature selection techniques such as recursive feature selection importantly considers both joint effects of SNPs and their possible interactions, identifying a set of SNPs with the best predictive performance [21, 22]. Combining machine learning (ML) with PRS has the potential to capture non-linear and complex interactions and facilitate better clinical decision-making. Thus, ML can complement existing statistical approaches to bring the prediction of disease or trait outcomes closer to clinical application.

In this study, rheumatoid arthritis (RA) was selected as a model disease to demonstrate the robustness of ML-optimized PRS in disease prediction. Affecting ~ 1% of the population worldwide, RA is one of the more prevalent chronic inflammatory joint diseases with mortality rates up to 54% higher than the general population [23, 24]. It is a complex autoimmune disease primarily characterised by the swelling of the joints leading to joint pain, stiffness and in severe cases irreversible joint damage. This is further exacerbated by several associated comorbidities such as coronary artery diseases and hyperlipidaemia [25]. Notably, as the onset of RA occurs in middle-aged adults at their economic and productivity prime, the effects of the disease poses a major socioeconomic burden on both patient and society [26, 27]. Hence, early identification of at-risk individuals may be critical in minimising the effects of the disease, through the provision of early preventive or mitigation measures or treatment. There is no single diagnostic test for RA and experts rely on patterns of clinical presentation.

Several non-genetic factors, including gender, smoking, pollutants, silica and asbestos have been found to modulate the risk of RA [28, 29]. The high prevalence of RA within families, with strongest risks observed in first-degree relative, suggests that genetics play an important role in RA development [30]. Since the first three RA GWAS were performed in 2007 [31], > 400 unique SNPs had been documented in the GWAS catalog (as of April 2022) [32] to be significantly associated with RA. While polymorphisms within HLA regions accounted for 11–37% of RA heritability and non-HLA risk loci were estimated to account for ~ 5% of heritability [33], > 50% of heritability remain unaccounted for [34]. Several studies have attempted to predict RA using known RA risk alleles (from previous association analyses) with some incorporating lifestyle and clinical characteristics (Additional file 2: Table S1).

**Fig. 1:**
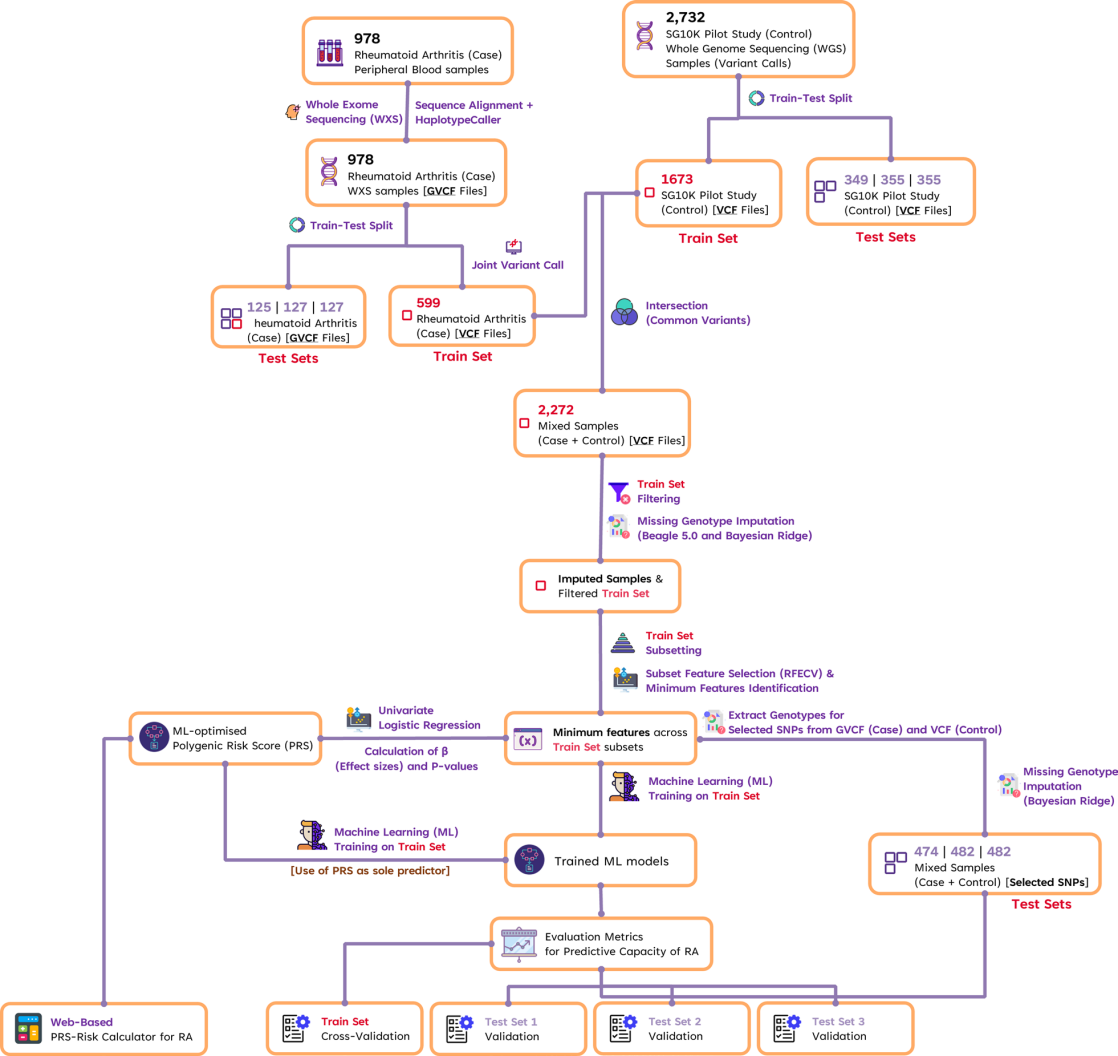
Summarised pipeline employed to identify predictors of RA. 978 RA case samples were split into a single training dataset (N = 599) and three test sets (N = 125/127/127). To maintain the ratio (61.2%/12.8%/13%/13%) between case and controls, the 2732 population control samples were similarly split in the same proportion, with a single training dataset (N = 1673), and three test sets (N = 349/355/355). Subsequently, the individual datasets were merged based on the common SNPs between both case and control datasets. The resultant training dataset was subjected to SNP filtering based on minor allele frequency genotype missingness or deviation from Hardy–Weinberg equilibrium. Missing genotypes were imputed using Beagle 5.0 initially and supplemented with machine learning imputation using the Bayesian Ridge algorithm. Training set was further divided into eight subsets of varying sample sizes prior to the implementation of recursive feature elimination with cross-validation (RFECV) using a Random Forest estimator. Commonly selected features following RFECV across the eight subsets were determined followed by stepwise inclusion of each of the commonly selected features based on their feature importance scores to identify the minimum number of features required to achieve an optimal performance metrics. The minimum features will then be determined as the final optimal feature set based on the evaluation of their predictive capacity across five diverse ML classifiers using cross-validation and separately in the three independent unseen test datasets. Likewise, a univariate logistic regression was used to establish the effect sizes of selected features for the calculation of the polygenic risk scores (PRS). PRS was also evaluated for its predictive capacity across the same five ML classifiers using cross-validation and separately in the three independent unseen test datasets. Finally, a PRS-Risk calculator for RA was developed to facilitate the calculation of PRS and RA-risk by providing the genotypes of the selected features of patients as inputs

Here, as a complement to GWAS, we employ ML, using similar algorithm that we previously reported for the prediction of methotrexate response in RA patients [35, 36], to select predictive SNPs that can robustly predict RA across 3 separate unseen test cohorts. We then developed an ML-optimized PRS to facilitate better clinical decision making. A summary of our overall ML strategy is presented in Fig. 1.

## Materials and methods

### Study cohort

This study examines 978 Singaporean RA patients of Chinese ethnicity, who are at least 18 years old, and satisfied the 1987 American College of Rheumatology revised criteria or the 2010 American College of Rheumatology/European League against Rheumatism criteria for RA. All protocols were performed according to the Declaration of Helsinki and written informed consent was collected from all participants. The study was approved by the National Healthcare Group Domain Specific Review Board (DSRB 2015/00582).

Whole-genome sequencing (WGS) data of 2732 Singaporean Chinese from the SG10K pilot study served as controls [37].

### Exome sequencing, sequence alignment, and quality control

The exome regions of genomic DNA, collected from peripheral blood mononuclear cells of 978 RA patients, were enriched using the Nimblegen SeqCap EZ kit (Roche). Exomes were captured using the Agilent SureSelect Human All Exon (V5/6) kit (Agilent Technologies, CA), followed by purification using AMPure XP system (Beckman Coulter, Beverly, USA). Quantification was subsequently performed using the Agilent high sensitivity DNA assay on the Agilent Bioanalyzer 2100 system. Whole exome sequencing was performed with Illumina HiSeq 4000 platform with 151 bp pair-end sequencing read.

### Training and test data

978 RA case samples were randomly split into a single training dataset (N = 599) and three test sets (N = 125/127/127). To maintain the ratio between case and controls, the 2732 population control samples were similarly split in the same proportion, with a single training dataset (N = 1673), and three test sets (N = 349/355/355). To ensure that the test cohort is truly ‘unseen’, samples were split into training and test datasets before further downstream analyses/processing.

### Sequence alignment, variant calling, and quality control

Utilising the BWA-MEM algorithm [38], the sequenced data of RA patients were aligned to the hs37d5 human reference genome, followed by the removal of duplicated reads using PICARD. Each sample was processed separately where realignment, recalibration and genotype calling were performed using the BaseRecalibrator and HaplotypeCaller modules of the Genome Analysis Toolkit (GATK). Using the genomicsDBImport and genotypeGVCF modules to call for variants on samples jointly [39] for the training dataset. For quality control, hard filtering of SNPs was performed based on GATK best practice (QD < 2.0, FS > 60.0, MQ < 40.0, SOR > 4.0, MQRankSum < − 12.5, ReadPosRankSum < − 8.0) using the VariantFiltration module.

### Pre-processing of Training dataset

Training dataset of both cases (N = 599) and controls (N = 1673) were merged together (N = 2272) using BCFtools [40] to identify SNPs that are common in both case and control datasets. The merged training dataset was further processed by removing SNPs with minor allele frequency < 1%, or > 10% genotype missingness or deviate from Hardy–Weinberg equilibrium (*p-value* < 0.01).

Missing genotypes were phased and imputed using the Beagle 5.1 software with HapMap Phase II recombination maps and 1000 Genomes Project phase III reference panels for each respective chromosome [41]. Thereafter, a Bayesian Ridge model, coupled with the IterativeImputer function from the Scikit-learn Python module [42], was fitted using the training dataset. This fitted model was then used to impute the remaining unimputed genotypes in the training dataset.

### Selecting features/SNPs that are predictive for RA cases

Within the training set, features identified to have the same genotype across > 90% of the samples were excluded from subsequent analyses. Additionally, for features sharing a > 95% correlation (Pearson Correlation Coefficient) in each chromosome, only one of the correlated features was retained for further analyses. The remaining training dataset of 76,713 SNPs was then processed into eight subsets of variable sample sizes using a stratified random sampling with replacement approach to ensure that features selected are stable. Thereafter, the recursive feature elimination with cross-validation (RFECV) algorithm was implemented with a Random Forest classifier estimator using the Scikit-learn Python module to identify an optimal set of important features sufficient for the prediction for each training subset. With the goal of obtaining features with a high stability of importance [35, 36, 43], features commonly selected across all eight subsets were chosen as the final set of features for further evaluation of predictive performances.

### Extraction of genotypes for selected SNPs for the test datasets

Using BCFtools, the genotype data for RA case samples in test sets were identified for the selected SNPs (after training) directly from the GVCF files produced from the HaplotypeCaller step. Separately, genotype data for population control samples were extracted from the VCF files obtained from the SG10K Pilot Study. For each of the 3 unseen test datasets, genotype data from the RA case and population control samples were combined. Thereafter, a Bayesian Ridge model, coupled with the IterativeImputer function from the Scikit-learn Python module [42], was fitted using the training dataset. The fitted model was subsequently used for the imputation of any missing genotypes in each of the 3 unseen test datasets. The individual test datasets consisting of both RA cases and population control samples were then independently used to evaluate the predictive performance of models that were trained using the train dataset.

### Evaluating the predictive performance of selected features using supervised ML

The selected features were assessed across five diverse ML classifiers: Logistic Regression, Support Vector Machines, Naïve Bayes, Random Forest and XGBoost. Within the training dataset, a fivefold cross-validation using stratified k-fold was performed for each of the five classifiers. Evaluation of predictive performance was conducted by referencing metrics such as the area under the curve (AUC) of a receiver operating characteristic (ROC) curve, sensitivity, specificity, accuracy scores, and average precision (area under a precision-recall curve). Similarly, the five classifiers were also fitted with the entire training datasets composed of the selected features and tested against the three independent unseen test datasets for their predictive performances based on the same metrics. The selected features were ranked based on their mean feature importance scores provided by the Random Forest estimator used in RFECV. To identify the minimum number of features required to achieve an optimal performance metrics (namely AUC, sensitivity, and specificity), the selected features were added one at a time to train models and evaluated for their predictive performance. The Shapley Additive exPlanations (SHAP) method [44] was adopted to explore the contribution of the selected features in the machine learning models for the classification of RA case samples, focusing on the Random Forest classifier that was initially used for the selection of predictive features.

To verify that the observed predictive performances by the selected features are not a random occurrence, the same number of features were randomly sampled (with replacement) from all the features in the training dataset prior to performing RFECV. These randomly sampled features were evaluated across all three unseen test sets and the results were used to plot a distribution of model performances using the ROC-AUC metric, across 1,000 iterations of sampling.

### Annotation and analysis of potential variant functions

Selected SNPs were annotated using ANNOVAR [45] and SNPNexus database [46], to identify their corresponding functional regions and genes. To obtain information regarding their potential functionality (e.g., transcription factor binding sites, miRNA binding sites, exonic splice enhancer/silencer (ESE/ESS), etc.), we referenced these SNPs against the pfSNP database resource [47], which has been updated to include information such as expression-associated SNPs or expression quantitative trait loci (eQTLs) [48, 49]. SNPs that were not predicted to be potentially functional were further interrogated for neighbouring pfSNPs in linkage disequilibrium (LD) (R^2^ > 0.8). The WGS data from the SG10K pilot study was used to identify pfSNPs in LD with these selected SNPs.

### machine-learning-optimized polygenic risk scores (PRS)

To improve interpretability and clinical applicability of the identified predictive SNPs for individual patients, a PRS was developed based on the ML-identified predictive SNPs. The effect sizes of these predictive SNPs were determined through univariate logistic regression analyses using PLINK [50], assuming additive effects of allele dosage, of all the samples in the training dataset. The following is the formula for calculating the PRS based on our 13 SNPs [51]:

$$PRS=\frac{{\sum }_{i}^{13}{S}_{i}\times {G}_{ij}}{P\times {M}_{j}}$$

For each SNP (*i*) within a sample (*j*), the product of the SNP’s effect size (*S*_*i*_) and the sample’s allelic dosage (*G*_*ij*_) was calculated. The resultant product for all selected SNPs were then summed and divided by the product of the ploidy (*P*) of an individual (2 for humans) and the number of non-missing variants in that sample (*M*_*j*_). The resultant PRS takes into consideration the possibility of missing genotypes by identifying the average PRS through the division of the number of non-missing SNP dosages. Most importantly, it prevents PRS of samples with missing genotypes to be consistently lower than those with complete data of their genotypes, mitigating bias of these samples towards a lower risk [52]. The distribution of PRS of samples in the training set was then plotted.

The same effect sizes of SNPs established from the training set was similarly used to calculate the PRS of samples across the 3 unseen test sets. Logistic regression was performed to examine the significance of association between the calculated PRS with RA. Using PRS as the sole predictor in our ML models, we further assessed the predictive capacity of PRS for RA.

## Results

### Case and population control datasets are comparable

Exome sequencing was performed on Singaporean Chinese RA case samples, while WGS data of Singaporean Chinese population controls were obtained from the SG10K pilot study [37]. As data from cases and controls were derived from different sequencing platforms, principal component analyses (PCA) were performed to establish that there was no batch effect that could confound our analysis (Additional file 1: Fig. S1).

### A signature of 9 SNPs was identified that robustly classifies RA in 3 independent unseen datasets

**Fig. 2:**
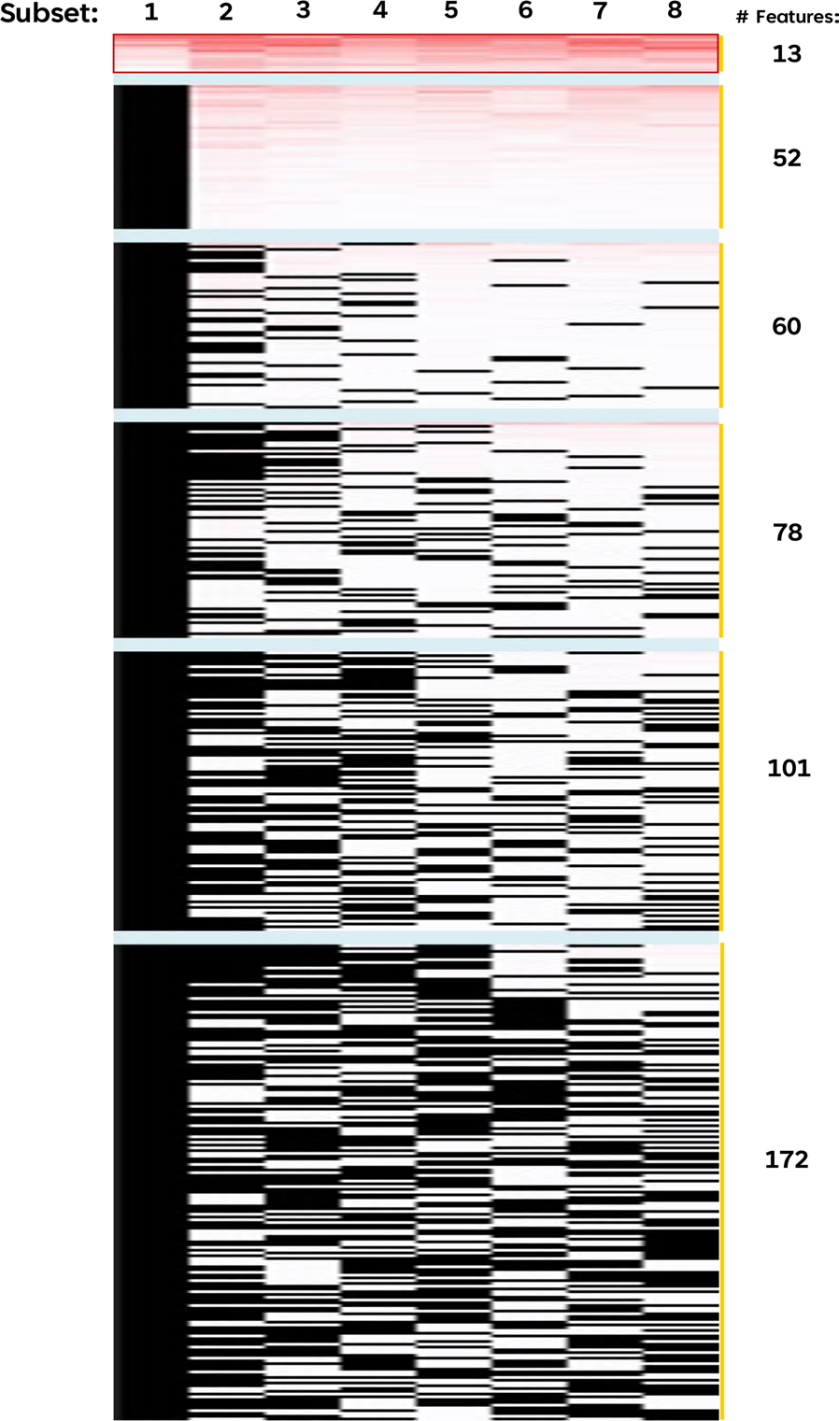
Number of important features (SNPs) identified across the eight training subsets of variable sample sizes from RFECV. Each column represents the different training subsets, and each row represents the individual features. Features are row-sorted based on the number of subsets that they were commonly identified in, with each block separated by a pale-blue divider (i.e., the first block of features, highlighted by a red box, represented the SNPs that were identified across all eight subsets based on the RFECV algorithm). Intensity of red represent the importance of the feature (based on the feature importance score) within each subset; Black represents features that were not identified to be important in the respective subset

**Fig. 3:**
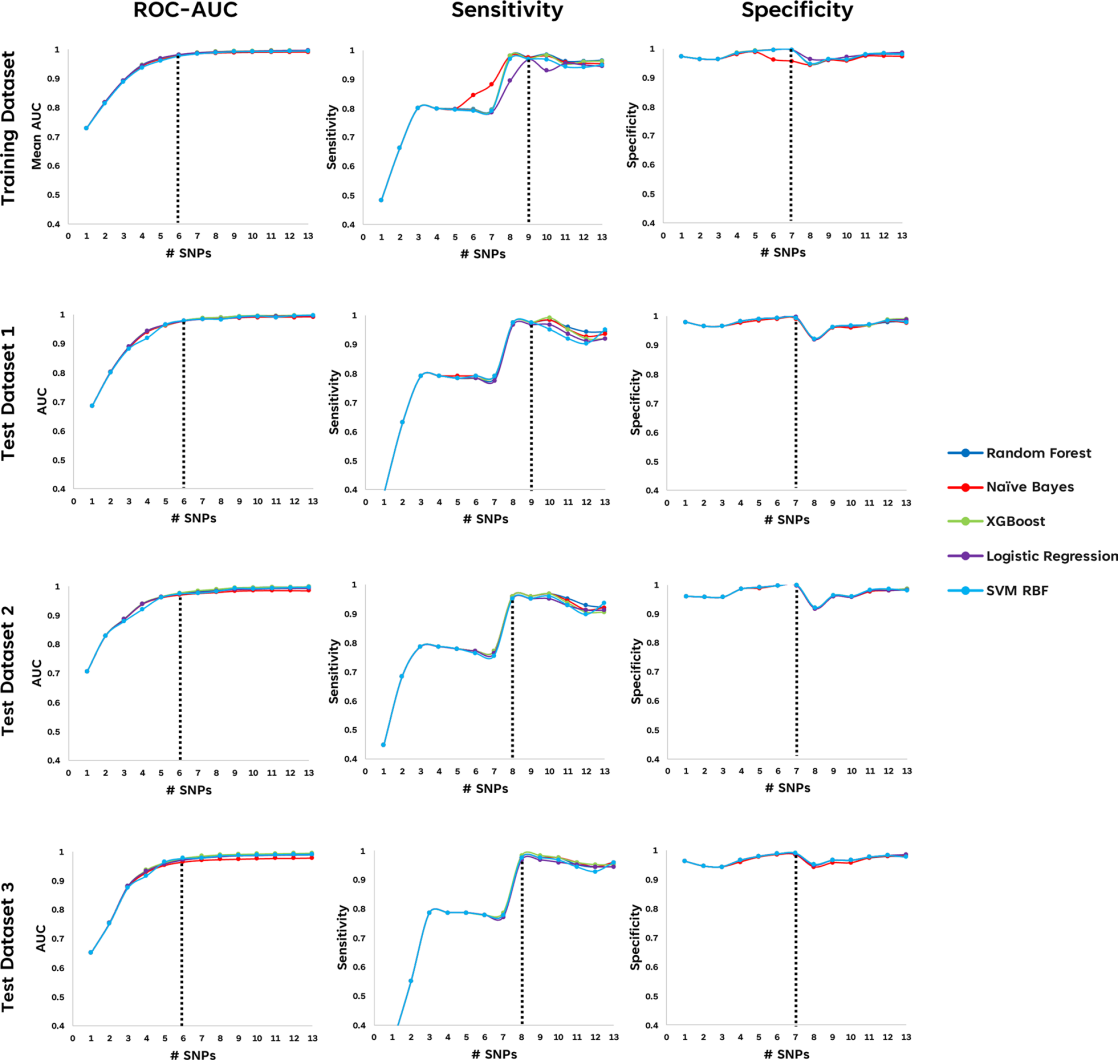
ROC-AUC, Sensitivity, and Specificity scores using an increasing number of the commonly selected SNPs from RFECV based on their mean feature importance scores for prediction of RA. Each of the commonly selected SNPs from RFECV were gradually included based their feature importance scores (from highest to lowest) in the evaluation of using fivefold cross-validation of training set, unseen test set 1, unseen test set 2, and unseen test set 3. Evaluation scores (ROC-AUC, Sensitivity, Specificity) were plotted against the number of selected SNPs (# SNPs) included in the prediction model. Dotted vertical line in each plot represented the determined optimal number of SNPs for a good evaluation score

To reduce dimensionality and identify a robust set of SNPs that are resistant to sample size bias [35, 36], feature selection using the RFECV algorithm was employed on eight randomly generated variable-sized sample subsets. Thirteen SNP features, with mean feature importance scores between 0.0118 and 0.0612 were commonly identified across all 8 subsets (Fig. 2). To identify the minimum number of features necessary for optimal predictive performance, stepwise inclusion of each of the 13 SNPs based on their feature importance scores (Additional file 2: Table S2) was assessed through cross-validation in the training dataset across all 5 ML models as well as the 3 independent unseen test datasets. As shown in Fig. 3, 9 out of the 13 SNPs (reduction of 30%) was required to achieve a reasonable predictive performance in all the 3 metrics examined (> 90% for AUC, sensitivity, and specificity) across both the training as well as 3 independent unseen test datasets. While 8 SNPs were sufficient to achieve reasonably good AUC and sensitivity, the addition of the 8th SNP resulted in a dip in the specificity, hence 9 SNPs is an optimal number to achieve high performance in both sensitivity and specificity, in addition to AUC.

**Fig. 4:**
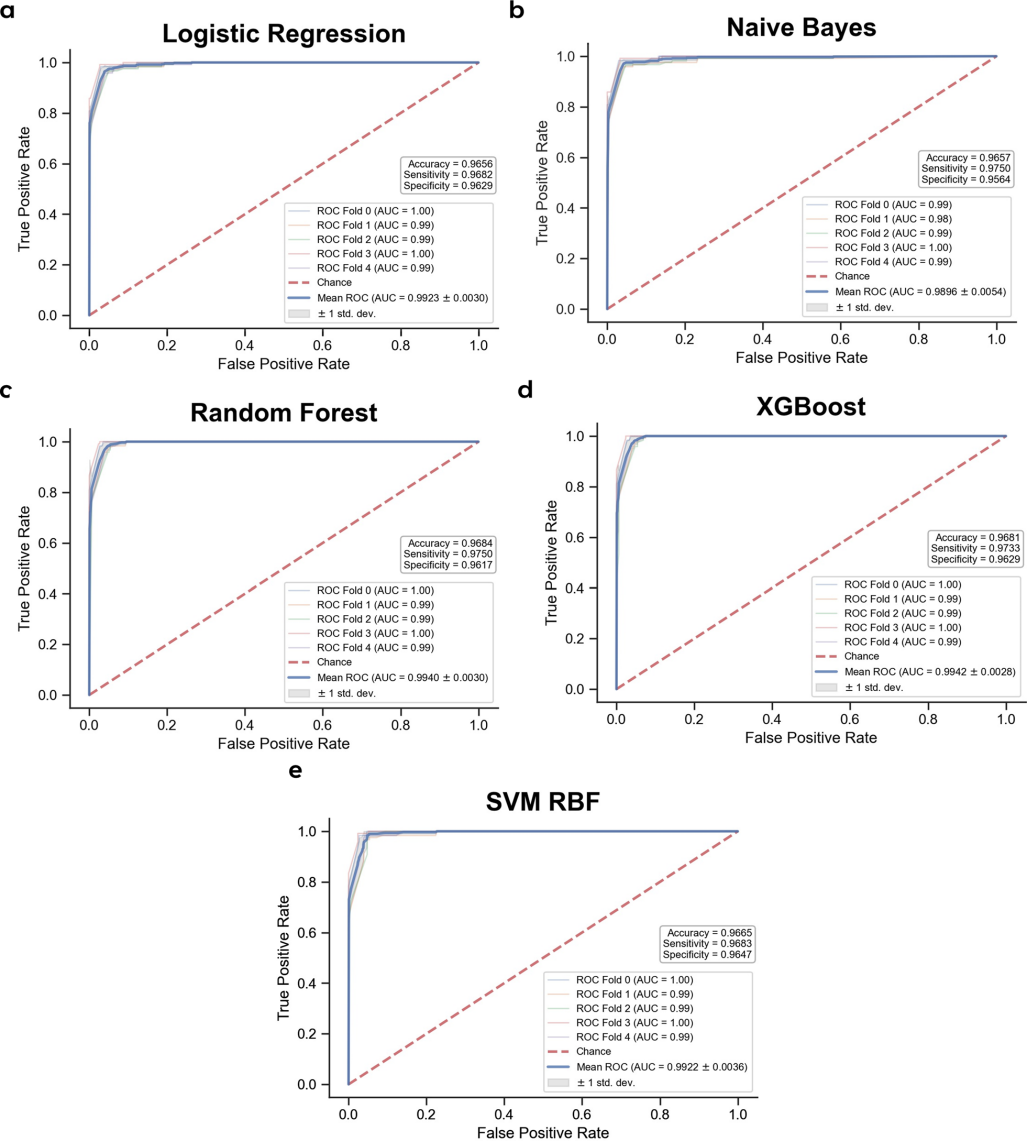
Predictive performance of 9 selected SNPs in the training set using fivefold cross-validation. ROC-AUC curves with Accuracy, Sensitivity, and Specificity of 9 selected SNPs using **a** Logistic regression, **b** Naïve bayes, **c** Random forest, **d** XGBoost, and **e** Support vector machine (SVM) classifiers

**Fig. 5:**
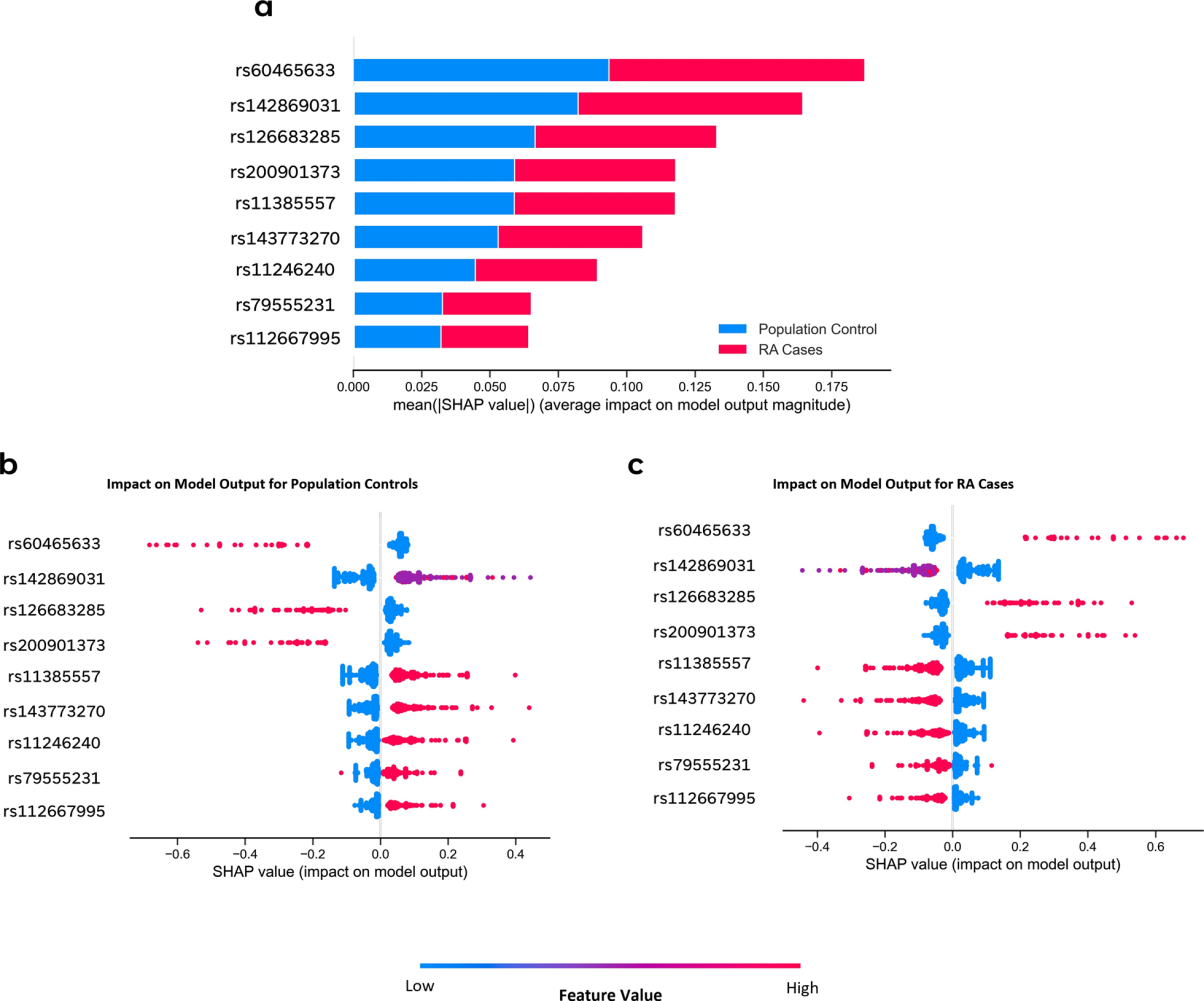
Summary of the impact of the 9 selected SNP features on Random Forest model output. Summary of the impact on the Random Forest classifier model output by the 9 selected SNP features. **a** Average impact on both model output (Population control and RA cases). **b** Impact on Population control classification based on feature values. **c** Impact on RA cases classification based on feature values

These 9 SNPs achieved mean AUC values between 0.990 and 0.994 when assessed using Cross-Validation in the Training dataset across all 5 selected ML models (Table 1, Fig. 4). Significantly, when tested against 3 independent unseen datasets, these same 9 SNPs performed exceptionally well, with AUC > 0.97 and all other pertinent metrics (F1 Score, Accuracy, Sensitivity,Specificity, and Average Precision) above 0.90 in all the different ML models (Table 2 and Additional file 1: Figures S2-S4, S6-S8). SHAP analyses of the 9 selected SNPs within the Random Forest classifier model (Fig. 5) reveals the contribution of each of the SNPs towards the model prediction output, ordered from the SNPs with the greatest contribution to the least amongst the 9 SNPs.

**Table 1:** Predictive performance of the 9 selected SNPs in a fivefold cross-validation of the Training dataset

**Dataset**	**Evaluation** **metric**	**Machine learning models**				
		**Logistic** **regression**	**Naïve** **bayes**	**Random** **forest**	**XGBoost**	**SVM RBF**
**Training set** **Cross-validation**	**Mean AUC**	0.992	0.990	0.994	0.994	0.992
	**Mean Sensitivity**	0.968	0.975	0.975	0.973	0.968
	**Mean Specificity**	0.963	0.956	0.962	0.963	0.965
	**Mean accuracy**	0.966	0.966	0.968	0.968	0.966
	**Mean average precision (PR-AUC)**	0.979	0.973	0.980	0.981	0.968

**Table 2:** Predictive performance of the 9 selected SNPs in each of the 3 unseen Test datasets

**Dataset**	**Evaluation** **metric**	**Machine learning models**				
		**Logistic** **regression**	**Naïve** **bayes**	**Random** **forest**	**XGBoost**	**SVM RBF**
**Test 1** **evaluation**	**AUC**	0.990	0.986	0.993	0.994	0.991
	**Sensitivity**	0.928	0.936	0.928	0.936	0.936
	**Specificity**	0.963	0.960	0.963	0.963	0.963
	**F1 score**	0.913	0.914	0.913	0.918	0.918
	**Accuracy**	0.945	0.948	0.945	0.949	0.949
	**Avg. Precision**	0.970	0.951	0.975	0.976	0.970
**Test 2** **evaluation**	**AUC**	0.988	0.982	0.989	0.992	0.990
	**Sensitivity**	0.937	0.945	0.945	0.945	0.937
	**Specificity**	0.961	0.961	0.963	0.963	0.963
	**F1 Score**	0.915	0.920	0.923	0.923	0.919
	**Accuracy**	0.949	0.953	0.954	0.954	0.950
	**Avg. Precision**	0.964	0.960	0.968	0.972	0.965
**Test 3** **evaluation**	**AUC**	0.987	0.972	0.987	0.991	0.986
	**Sensitivity**	0.913	0.921	0.921	0.937	0.921
	**Specificity**	0.966	0.958	0.966	0.966	0.966
	**F1 Score**	0.910	0.903	0.914	0.922	0.914
	**Accuracy**	0.940	0.940	0.944	0.952	0.944
	**Avg. Precision**	0.961	0.936	0.958	0.965	0.946

**Fig. 6:**
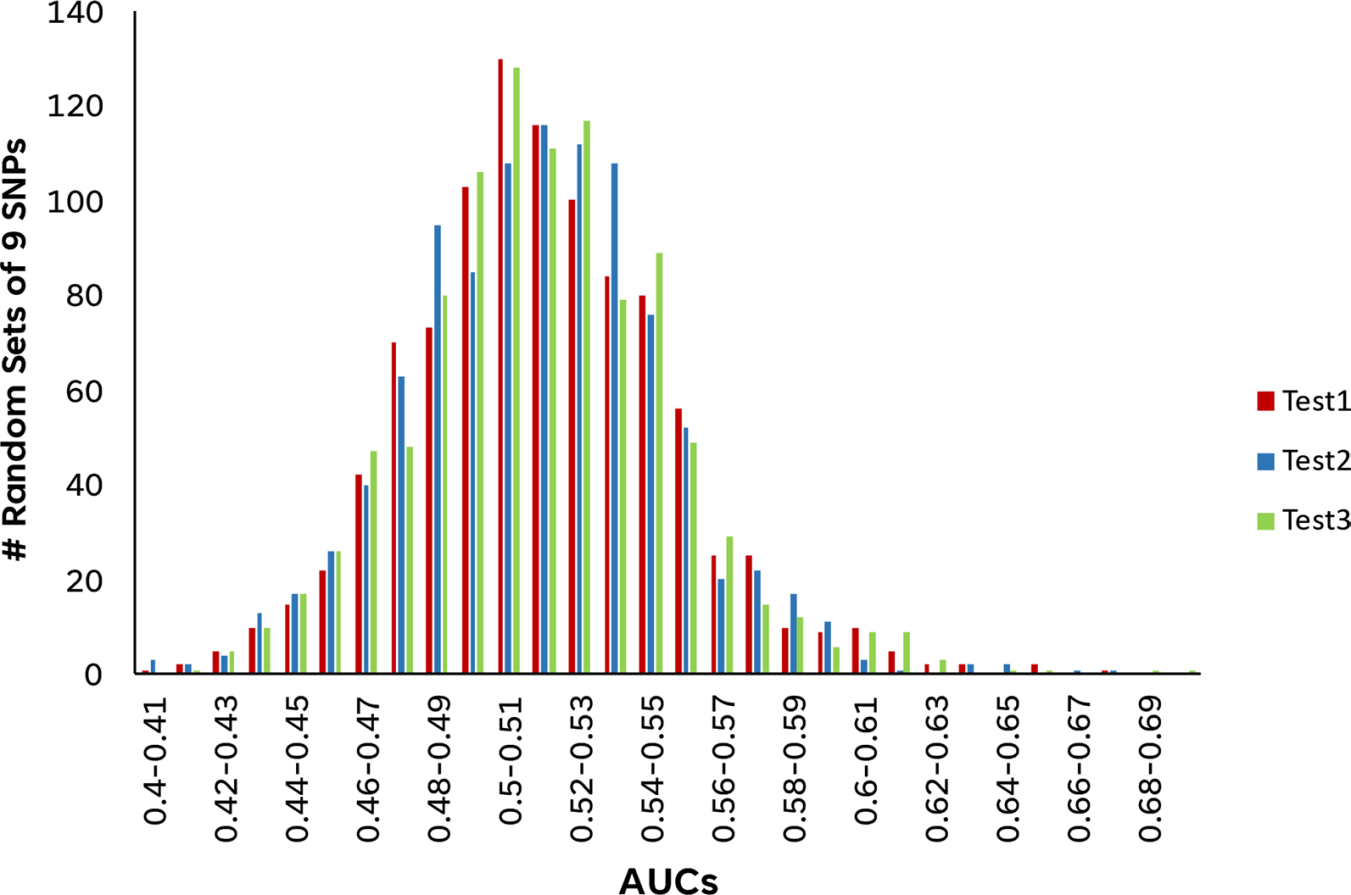
Distribution of AUC scores obtained from 1000 sets of randomly selected 9 SNPs. Distribution of AUC-ROC scores binned in intervals of 0.01 of 1000 sets of randomly selected 9 SNPs to verify that predictive performance observed from the selected 9 SNPs by the feature selection pipeline employed was a non-random occurrence. AUC-ROC scores were obtained by evaluation in each of the three unseen test sets using one of the five chosen ML classifiers, the Random Forest classifier

With such excellent predictive performance in 3 different unseen datasets, it is pertinent to evaluate the validity of the observation and give assurance that the excellent predictive performance is not merely due to random chance. A thousand iterations of random sampling of 9 SNPs from the total pool of > 70,000 SNPs were performed. These randomly selected 9 SNPs were then evaluated, as above, for predictive performance using Random Forest, one of the 5 ML models, in the 3 unseen Test dataset. AUCs obtained were then binned with intervals of 0.01 and the distribution of AUCs were plotted. As evident in Fig. 6, the AUCs of the 1000 randomly identified 9 SNPs are normally distributed with peak AUC between 0.50 and 0.51 and the highest AUC is less than 0.7.

### PRS utilising 9 ML-identified predictive SNPs clearly distinguishes RA patients from healthy individuals

**Fig. 7:**
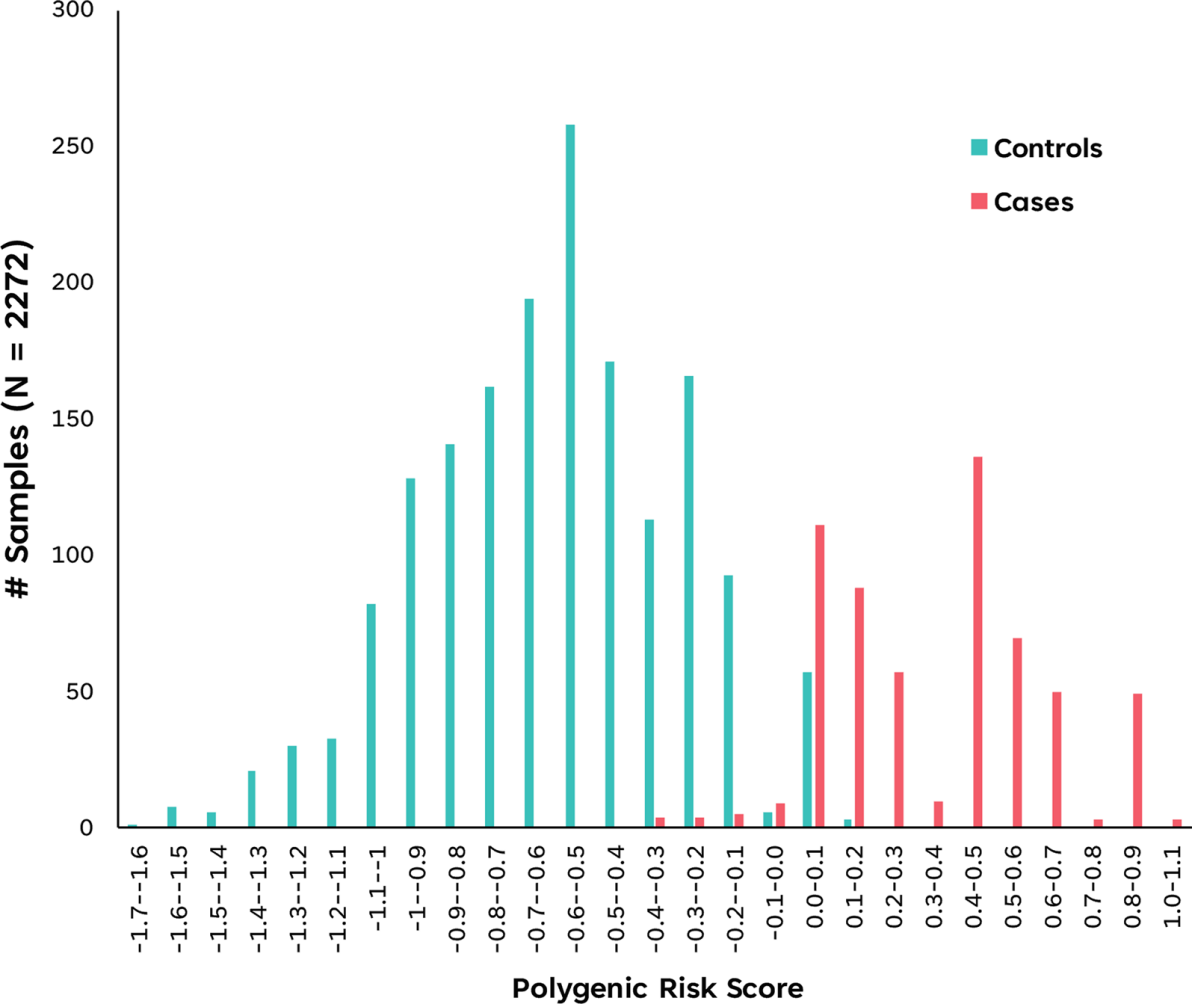
Distribution of Polygenic Risk Scores (PRS) of samples within Training dataset. Distribution plots of PRS scores binned in intervals of 0.1 for RA case samples and Population control samples

**Fig. 8:**
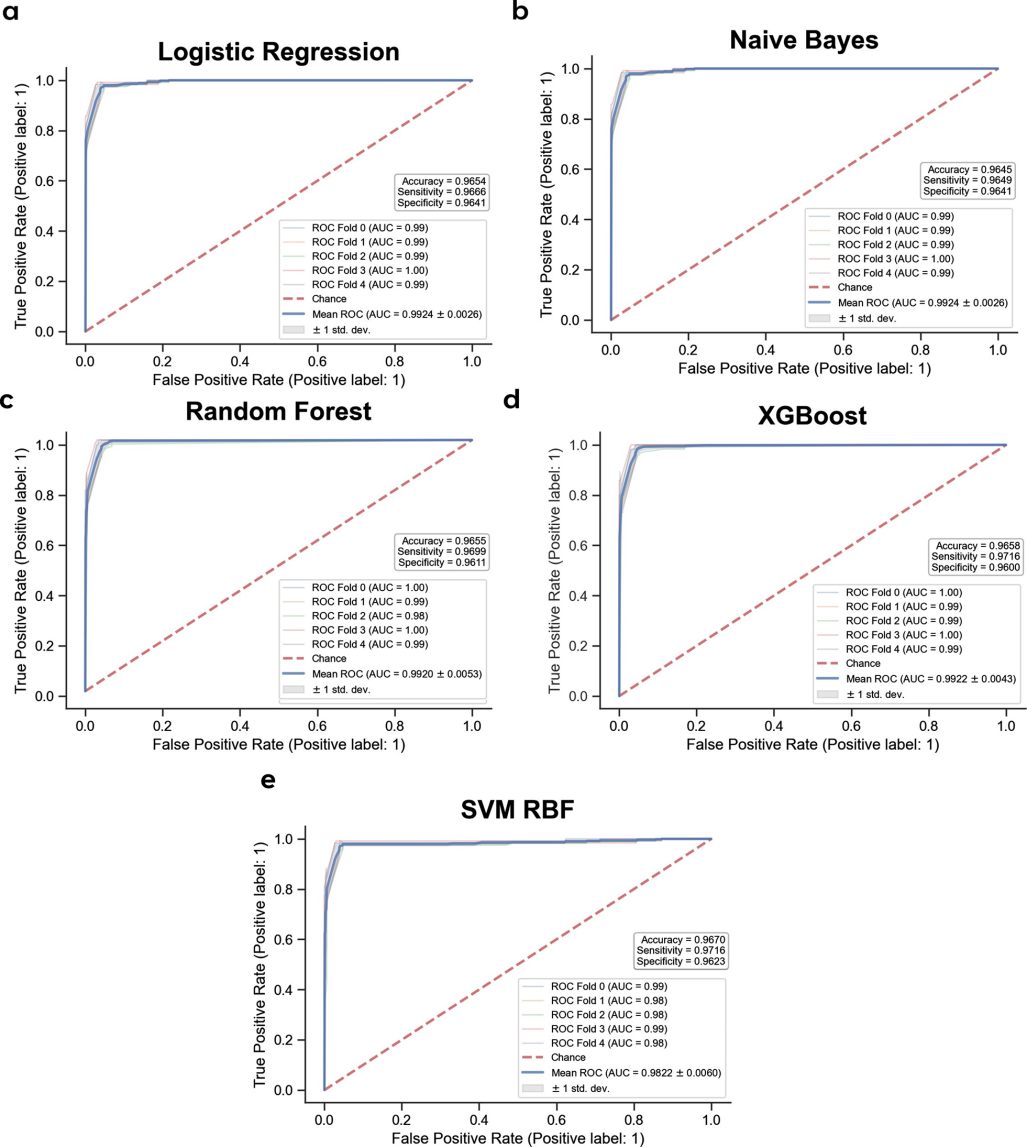
Predictive performance of using PRS in the training set using fivefold cross-validation. ROC-AUC curves with Accuracy, Sensitivity, and Specificity of PRS using **a** Logistic Regression, **b** Naïve Bayes, **c** Random Forest, **d** XGBoost, and **e** Support Vector Machine (SVM) classifiers

Univariate logistic regression analyses revealed that all 9 ML-identified predictive SNPs were significantly associated with RA (P < 1 × 10^–5^) (Additional file 2: Table S2). Six ML-identified predictive SNPs have effect sizes in the negative range (β: − 5.24 to − 2.23) and hence confer protection against RA while the remaining three SNPs with positive effect sizes (β: 3.50to 7.28) predispose to RA. The ML-optimized PRS of training set samples from RA patients and control population displayed relatively normal but distinct distributions with some overlap (− 0.3 to 0.2), with PRS of RA patients ranging from − 0.3 to 1.1, while PRS of control population ranges from − 1.7 to 0.2 (Fig. 7). Notably, using logistic regression analyses, PRS was found to be significantly associated (P < 1 × 10^–6^) across all 3 unseen test sets. Significantly, the predictive performance of the sole ML-optimized PRS (Additional file 2: Table S5, Fig. 8 and Additional file 1: Figures S9–S11) was found to be comparable to ML-identified 9 SNPs (Tables 1 and 2) across all 5 selected ML models and 3 independent test sets.

### Characteristics of these 9 Predictive SNPs

Although these 9 SNPs were identified primarily from exome sequenced DNA, the majority (6, 67%) of these SNPs are intronic, while 3 (33%) reside within exons (Additional file 2: Table S2). The 3 exonic SNPs were all non-synonymous, with one predicted to be a deleterious alteration. Two of the intronic predictive SNPs are potential eQTL (expression quantitative trait locus) SNPs, predicted to be associated with changes in expression (Additional file 2: Table S2). Of the other 4 predictive intronic SNPs, 3 SNPs are in strong linkage disequilibrium (LD) (R^2^ > 0.8) with SNPs that are potentially functional (Additional file 2: Table S3).

To gain further insight into the significance of these predictive SNPs, several GWAS databases were interrogated to determine if any of these SNPs were previously reported to be significantly associated with any phenotype. Three SNPs were identified by 3 GWAS databases (BioBank Japan PheWeb [53], IEU Open GWAS Project [54], GWAS Atlas [55]) to be significantly (P < 1 × 10^–5^) associated with various phenotypes (Additional file 2: Table S4). These phenotypes were summarized into 7 general categories (Additional file 2: Table S2).

The 9 genic SNPs reside in 10 different genes with one residing in the exonic regions of 2 genes. These genes reside in pathways such as signal transduction, sensory perception, immune, and metabolism of lipids/proteins (Additional file 2: Table S2). Amongst these pathways, some such as signal transduction, sensory perception, immune, metabolism of lipids/proteins are consistent with characteristics of pathology/development of RA. Two of these genes have previously been reported to be associated with RA (Additional file 2: Table S2).

## Discussion

In this study, through rigorous ML feature selection that is tolerant to differences in sample sizes [35, 36], we identified a signature of 9 SNPs that can predict RA with excellent predictive performance not only in the training dataset (mean AUC > 0.99; mean sensitivity > 0.96, mean specificity > 0.95, mean accuracy > 0.96, and mean average precision > 0.96) assessed through cross-validation, but also in not one, but 3 independent, unseen test datasets (AUC > 0.97; sensitivity > 0.91, specificity > 0.95, F1 score > 0.90, accuracy > 0.94, and average precision > 0.93 in all 3 test datasets). This excellent predictive performance is unlikely due to random chance since the predictive performance of 1,000 9 randomly selected SNPs was poor with AUC < 0.7, and majority of the 9 randomly selected SNPs have AUC of only between 0.50 and 0.51.

To facilitate interpretability and clinical applicability of the 9 ML-identified predictive SNPs for individual patients, a PRS was developed based on these 9 ML-identified predictive SNPs. Notably, not only are the 9 ML-identified predictive SNPs significantly (P < 1 × 10^–5^) associated with RA individually, the calculated PRS score (from these 9 SNPs) were also found through logistic regression to be significantly (P < 1 × 10^–6^) associated with RA across all 3 unseen datasets and this single ML-optimized PRS was also found to have comparable excellent predictive performance as the 9 SNPs across all 5 selected ML models and 3 independent test sets. To further facilitate the potential clinical application, an RA ML-optimized PRS calculator based on the 9 ML-identified predictive SNPs was developed; it is accessible via this link: https://xistance.shinyapps.io/prs-ra/. In this RA ML-optimized PRS calculator, the genotype of the 9 ML-identified predictive SNPs in a patient are entered and the PRS, odds as well as probability of the patient developing RA will be given, enabling the healthcare provider to make an earlier diagnosis when the patient presents with only minimal clinical signs.

Although exome sequencing data was interrogated, only a minority (3/9) of the predictive SNPs reside in the coding region. Two (rs1266832853 and rs60465633) of the 3 coding predictive SNPs are benign, non-synonymous susceptibility SNPs with positive effect sizes while one (rs143773270) is a potential deleterious, non-synonymous protective SNP with negative effect size. The majority of the predictive SNPs resides in the introns (6/9). Most (5/6) of these intronic predictive SNPs are potentially protective SNPs with negative effect sizes. These potentially protective predictive SNPs in non-coding regions with negative effect sizes, are potential eQTL SNPs or are in strong LD (R^2^ > 0.8) with non-interrogated SNPs that are predicted to be potentially functional, modulating expression of the gene by being eQTL SNPs, or potentially altering transcription factor binding sites (TFBS) or intronic splicing regulatory elements (ISRE). The putative functionality of the sole intronic predictive susceptibility SNP (rs200901373) with positive effect size remains unknown. These data thus suggest that while the majority (2/3) of the non-synonymous, predictive coding SNPs have positive effect size and thus may confer susceptibility to RA through modulating protein structure/function, the majority of the (5/6) of intronic predictive SNPs have negative effect size and thus may confer protection against RA through modulating the expression of the intricate network of genes as these intronic predictive SNPs are either eQTL SNPs or have potential to alter TFBS/ISRE sites. Validating the potential function and effect of these SNPs in the RA pathway would be a worthwhile future direction.

None of the 9 predictive SNPs have previously been reported to be associated with RA. To gain further insight into these 9 predictive SNPs, numerous GWAS databases were interrogated to evaluate if any of these 9 predictive SNPs were previously reported via GWAS to be associated with any disease/phenotype/function which may help explain the role of these SNPs/genes in RA. As GWAS mainly interrogate tag-SNPs and this study interrogates exomic SNPs, only 3 of these 9 predictive SNPs were reported by 3 GWAS databases (BioBank Japan PheWeb [53], IEU Open GWAS Project [54], GWAS Atlas [55]) to be significantly associated (P < 1 × 10^–5^) (Additional file 2: Table S4) with several different functions (including eQTL association) and some diseases, which can be categorized into 6 different themes (Additional file 2: Table S2). Notably, most of these association were consistent with the characteristics or phenotype of RA. For example, rs11385557 in the intronic region of BAIAP2L1 was found in OpenGWAS database to be significantly (P < 7.69 × 10^–5^) associated with peripheral nerve disorders (Additional file 2: Table S4, #8) which is consistent with RA patients often experiencing peripheral neuropathy with pain, numbness, and muscle weakness [56]. Similarly, rs11385557 was also reported by OpenGWAS database to be significantly (P < 9.08 × 10^–5^) associated with lymphocyte and monocyte counts (Additional file 2: Table S4, #1–4). This is consistent with reports of lymphopenia (low lymphocyte counts) commonly observed in RA patients [57], as well as monocytes activation and migration into joints in early RA [58]. Hence, it may be worthwhile to further investigate their roles in RA.

Although these 9 SNPs were not previously associated with RA, 2 of them reside in disease susceptibility genes (Additional file 2: Table S2, Row 5 and 9). rs11385557 is found in the intron of the BAIAP2L1, an insulin receptor tyrosine kinase gene (Additional file 2: Table S2). The expression of the BAIAP2L1 gene is positively correlated with C-reactive protein (CRP) levels within fibroblast-like synovial cells from RA patients [61]. CRP is an immune regulator that is commonly used as a marker for systemic inflammation in RA [62]. Although the intronic SNP was not predicted to be potentially functional, it was found to be in strong linkage disequilibrium (r^2^ > 0.8) with 19 potentially functional SNPs, most of which are associated with modulation of gene expression (eQTL SNPs), while some are predicted to alter intronic splice regulatory elements (ISRE) (Additional file 2: Table S3). Hence, the above observation is consistent with SNPs in LD with rs11385557 modulating gene expression of the BAIAP2L1 gene, which in turn alters the CRP levels in RA patients. rs112667995 resides within the intron of the PRKN (Parkin RBR E3 Ubiquitin Protein Ligase) gene. PRKN deficiency ameliorates inflammatory arthritis through the suppression of p53 degradation [63]. Similarly, although this intronic SNP (rs79555231) is not predicted to be potentially functional (Additional file 2: Table S2), it is in strong LD (r^2^ > 0.8) with 21 potentially functional SNPs that are mainly predicted to alter transcription factor binding sites (TFBS) (Additional file 2: Table S3). Thus, SNPs in LD with rs79555231 may influence the expression of PRKN which in turn modulates inflammatory arthritis. Taken together, both these intronic SNPs are in LD with markers that modulate gene expression of either BAIAP2L1 or PRKN that is associated with RA. Since majority of the predictive SNPs are in non-coding regions and many of the potentially functional SNPs likely reside beyond the regions that were sequenced through exome sequencing, it may thus be worthwhile to build models from WGS data.

While these 9 SNPs displayed excellent predictive performance for RA in Singaporean Chinese population, future work could explore the generalizability of the predictive performance of these 9 SNPs in other populations. It may be worthwhile to initially determine whether these SNPs exhibit population differentiation between Singaporean Chinese and another population [64] before general adoption. Further studies could also focus on the characterization of the roles of the predictive coding SNPs in conferring predisposition to RA as well as the roles of the predictive intronic SNPs in conferring protection against RA.

These 9 SNPs have potential to be clinically applicable for diagnosing individual patients with RA through the development of rapid genotyping assays for these SNPs. With improvements of WGS technology coupled with its rapidly declining cost, it will not be surprising that most, if not all, individuals will have their genome sequenced in the foreseeable future. Then, it will be cost-effective to deploy PRS on a large scale. However, due to resource constraints, current WGS data is primarily stored as variant call format (VCF) files [65] with small data storage space requirements. One limitation of storing WGS data as VCF is that, in order to generate VCF files, raw sequences from a group of individuals are aligned and variants are then identified based on a reference genome. The sequence identity of loci which do not show variability between the group of individuals and the reference genome are not stored. As such, depending on the size of the group of individuals, the number of variations stored will be different, with more variation stored in VCF files of large and more diverse group and less variation stored in VCF files of smaller and more homogenous group of individuals. For locus without sequence identity assigned, it is not possible to accurately extract the genotype information at the individual level. One cannot assume that locus to be the homozygous genotype of the reference genome as the unassigned region could also be excluded due to low mapping quality or poor read coverage during sequencing. Hence, for WGS data to be clinically applicable at an individual level, an alternative VCF file, the genomic VCF (gVCF) file format which can be generated using the GATK suite [66], as done in this study, could be explored. Although the storage size required for gVCF files is larger than typical VCF files, it is still overall much smaller compared to the storage of BAM files containing the sequencing read alignments [67]. The advantage of the gVCF file is that it stores not only information of the genotypes of variant site, but it also compactly stores information of the invariant genomic regions, facilitating the more accurate assignment of genotype at invariant sites for clinical implementation of our prediction models.

## Conclusions

In summary, PRS of the 9 ML-selected predictive SNPs is significantly associated (P < 1 × 10^–6^) with and predictive (AUC > 0.9) of RA in all 3 independent, unseen test datasets. To facilitate individualized clinical applicability, RA ML-PRS calculator of these 9 SNPs (https://xistance.shinyapps.io/prs-ra/) was developed. Majority of the predictive SNPs are protective and reside in non-coding regions and are either predicted potentially functional SNPs (pfSNPs) or in high linkage disequilibrium (r2 > 0.8) with un-interrogated pfSNPs. These data highlight the promise of this ML strategy to identify useful genetic features that can robustly predict disease with good potential for clinical application.

## Supplementary Information

**Additional file 1:** **Figure S1–Figure S15.** **Figure S1.** Principal Component Analysis (PCA) of Case and Control samples from WXS and WGS respectively for the evaluation of underlying batch effects. (a) Plot of % of variance explained by each principal component (PC). (b) PCA plot of the first two principal components of variation based on the combined case and control samples from differing data sources. (c) Boxplots of the case and control samples within PC1. (d) Boxplots of the case and control samples within PC2. **Figure S2.** Predictive performance of 9 selected SNPs in unseen Test Set 1. ROC-AUC curves with F1 score, Accuracy, Sensitivity, and Specificity of 9 selected SNPs using (a) Logistic Regression, (b) Naïve Bayes, (c) Random Forest, (d) XGBoost, and (e) Support Vector Machine (SVM) classifiers. **Figure S3.** Predictive performance of 9 selected SNPs in unseen Test Set 2. ROC-AUC curves with F1 score, Accuracy, Sensitivity, and Specificity of 9 selected SNPs using (a) Logistic Regression, (b) Naïve Bayes, (c) Random Forest, (d) XGBoost, and (e) Support Vector Machine (SVM) classifiers. **Figure S4.** Predictive performance of 9 selected SNPs in unseen Test Set 3. ROC-AUC curves with F1 score, Accuracy, Sensitivity, and Specificity of 9 selected SNPs using (a) Logistic Regression, (b) Naïve Bayes, (c) Random Forest, (d) XGBoost, and (e) Support Vector Machine (SVM) classifiers. **Figure S5.** Predictive performance of 9 selected SNPs in training set using fivefold cross-validation. Precision-Recall curves of 9 selected SNPs using (a) Logistic Regression, (b) Naïve Bayes, (c) Random Forest, (d) XGBoost, and (e) Support Vector Machine (SVM) classifiers. **Figure S6.** Predictive performance of 9 selected SNPs in unseen Test Set 1. Precision-Recall curves with of 9 selected SNPs using (a) Logistic Regression, (b) Naïve Bayes, (c) Random Forest, (d) XGBoost, and (e) Support Vector Machine (SVM) classifiers. **Figure S7.** Predictive performance of 9 selected SNPs in unseen Test Set 2. Precision-Recall curves with of 9 selected SNPs using (a) Logistic Regression, (b) Naïve Bayes, (c) Random Forest, (d) XGBoost, and (e) Support Vector Machine (SVM) classifiers. **Figure S8.** Predictive performance of 9 selected SNPs in unseen Test Set 3. Precision-Recall curves with of 9 selected SNPs using (a) Logistic Regression, (b) Naïve Bayes, (c) Random Forest, (d) XGBoost, and (e) Support Vector Machine (SVM) classifiers. **Figure S9.** Predictive performance of calculated PRS in unseen Test Set 1. ROC-AUC curves with F1 score, Accuracy, Sensitivity, and Specificity of PRS using (a) Logistic Regression, (b) Naïve Bayes, (c) Random Forest, (d) XGBoost, and (e) Support Vector Machine (SVM) classifiers. **Figure S10.** Predictive performance of calculated PRS in unseen Test Set 2. ROC-AUC curves with F1 score, Accuracy, Sensitivity, and Specificity of PRS using (a) Logistic Regression, (b) Naïve Bayes, (c) Random Forest, (d) XGBoost, and (e) Support Vector Machine (SVM) classifiers. **Figure S11.** Predictive performance of calculated PRS in unseen Test Set 3. ROC-AUC curves with F1 score, Accuracy, Sensitivity, and Specificity of PRS using (a) Logistic Regression, (b) Naïve Bayes, (c) Random Forest, (d) XGBoost, and (e) Support Vector Machine (SVM) classifiers. **Figure S12.** Predictive performance of calculated PRS in the training set using fivefold cross-validation. Precision-Recall curves of PRS using (a) Logistic Regression, (b) Naïve Bayes, (c) Random Forest, (d) XGBoost, and (e) Support Vector Machine (SVM) classifiers. **Figure S13.** Predictive performance of calculated PRS in unseen Test Set 1. Precision-Recall curves of PRS using (a) Logistic Regression, (b) Naïve Bayes, (c) Random Forest, (d) XGBoost, and (e) Support Vector Machine (SVM) classifiers. **Figure S14.** Predictive performance of calculated PRS in unseen Test Set 2. Precision-Recall curves of PRS using (a) Logistic Regression, (b) Naïve Bayes, (c) Random Forest, (d) XGBoost, and (e) Support Vector Machine (SVM) classifiers. **Figure S15.** Predictive performance of calculated PRS in unseen Test Set 3. Precision-Recall curves of PRS using (a) Logistic Regression, (b) Naïve Bayes, (c) Random Forest, (d) XGBoost, and (e) Support Vector Machine (SVM) classifiers.

**Additional file 2: Table S1–Table S5. Table S1.** Summary of studies that have used of genetics in the prediction of RA. **Table S2.** Detailed information of the 9 selected SNPs and their genes from feature selection. **Table S3.** Potentially functional SNPs (pfSNPs) in linkage disequilibrium with selected SNPs without previously established potential function. **Table S4.** Previously identified GWAS associations of selected SNPs at p-value significance of 1 × 10^−5^ from BioBank Japan PheWeb, IEU Open GWAS Project, and GWAS Atlas. **Table S5.** Predictive performance of Polygenic Risk Scores (PRS) calculated from the 9 selected SNPs in a fivefold cross-validation of the Train dataset and in each of the 3 unseen Test datasets.

## Data Availability

The data that support the findings of this study are available from the corresponding author, but restrictions apply to the availability of these data, which were used under the license for the current study, and so are not publicly available. Data are however available from the authors upon reasonable request and with permission of the corresponding author.

## Abbreviations

ML: Machine-learning
PRS: Polygenic risk scores
RA: Rheumatoid arthritis
SNP: Single nucleotide polymorphisms
pfSNPs: Potentially functional single nucleotide polymorphisms
GWAS: Genome wide association studies
LD: Linkage disequilibrium
RPVT: Raw p-value thresholding
WGS: Whole-genome sequencing
GATK: Genome Analysis Toolkit
RFECV: Recursive feature elimination with cross-validation
ESE/ESS: Exonic splice enhancer/silencer
eQTLs: Expression quantitative trait loci
PCA: Principal component analysis
AUC-ROC: Area under the curve of a receiver operating characteristic curve
TFBS: Transcription factor binding site
ISRE: Intronic splicing regulatory elements
VCF: Variant call format

## Code availability

The codes that support the findings of this study are available from the corresponding author, but restrictions apply to the availability of these codes, which were used under the license for the current study, and so are not publicly available. Codes are however available from the authors upon reasonable request and with permission of the corresponding author.
