# Supplementary material for: Robust SNP-based prediction of rheumatoid arthritis through machine-learning-optimized polygenic risk score
Source: J Transl Med. 2023 Feb 7;21:92. doi: 10.1186/s12967-023-03939-5 (PMC9903430; doi:10.1186/s12967-023-03939-5)
Supplement: Supplementary file 1 — Additional file 1: Figure S1–Figure S15. Figure S1. Principal Component Analysis (PCA) of Case and Control samples from WXS and WGS respectively for the evaluation of underlying batch effects. (a) Plot of % of variance explained by each principal component (PC). (b) PCA plot of the first two principal components of variation based on the combined case and control samples from differing data sources. (c) Boxplots of the case and control samples within PC1. (d) Boxplots of the case and control samples within PC2. Figure S2. Predictive performance of 9 selected SNPs in unseen Test Set 1. ROC-AUC curves with F1 score, Accuracy, Sensitivity, and Specificity of 9 selected SNPs using (a) Logistic Regression, (b) Naïve Bayes, (c) Random Forest, (d) XGBoost, and (e) Support Vector Machine (SVM) classifiers. Figure S3. Predictive performance of 9 selected SNPs in unseen Test Set 2. ROC-AUC curves with F1 score, Accuracy, Sensitivity, and Specificity of 9 selected SNPs using (a) Logistic Regression, (b) Naïve Bayes, (c) Random Forest, (d) XGBoost, and (e) Support Vector Machine (SVM) classifiers. Figure S4. Predictive performance of 9 selected SNPs in unseen Test Set 3. ROC-AUC curves with F1 score, Accuracy, Sensitivity, and Specificity of 9 selected SNPs using (a) Logistic Regression, (b) Naïve Bayes, (c) Random Forest, (d) XGBoost, and (e) Support Vector Machine (SVM) classifiers. Figure S5. Predictive performance of 9 selected SNPs in training set using fivefold cross-validation. Precision-Recall curves of 9 selected SNPs using (a) Logistic Regression, (b) Naïve Bayes, (c) Random Forest, (d) XGBoost, and (e) Support Vector Machine (SVM) classifiers. Figure S6. Predictive performance of 9 selected SNPs in unseen Test Set 1. Precision-Recall curves with of 9 selected SNPs using (a) Logistic Regression, (b) Naïve Bayes, (c) Random Forest, (d) XGBoost, and (e) Support Vector Machine (SVM) classifiers. Figure S7. Predictive performance of 9 selected SNPs in unseen Test Set 2. P [file 12967_2023_3939_MOESM1_ESM.docx]

**Robust SNP-based prediction of Rheumatoid Arthritis through Machine-Learning- Optimized Polygenic Risk Score.**

Ashley J.W. Lim^1^, Tyniana C. Tera^2^, Lee Jin Lim^1^, Justina Wei Lynn Tan^3^, Ee Tzun Koh^3^, TTSH Rheumatoid Arthritis Study Group^3^**,** Samuel S. Chong^4^, Chiea Chuen Khor^5^, Khai Pang Leong^3,9^, Caroline G. Lee^1,6,7,8*^

^*^ Corresponding author’ emails: [bchleec@nus.edu.sg](mailto:bchleec@nus.edu.sg)

**This file includes:**

[**Supplementary Methods** 3](#_Toc123135999)

[**Figure S1** 4](#_Toc123136000)

[**Figure S2** 5](#_Toc123136001)

[**Figure S3** 6](#_Toc123136002)

[**Figure S4** 7](#_Toc123136003)

[**Figure S5** 8](#_Toc123136004)

[**Figure S6** 9](#_Toc123136005)

[**Figure S7** 10](#_Toc123136006)

[**Figure S8** 11](#_Toc123136007)

[**Figure S9** 13](#_Toc123136008)

[**Figure S10** 14](#_Toc123136009)

[**Figure S11** 15](#_Toc123136010)

[**Figure S12** 16](#_Toc123136011)

[**Figure S13** 17](#_Toc123136012)

[**Figure S14** 18](#_Toc123136013)

[**Figure S15** 19](#_Toc123136014)

[**Table S1** 20](#_Toc123136015)

[**Table S2** 20](#_Toc123136016)

[**Table S3** 20](#_Toc123136017)

[**Table S4** 20](#_Toc123136018)

[**Table S5** 20](#_Toc123136019)

# **Supplementary Methods**

**Verification of underlying ‘Batch Effects’ using Principal Component Analysis (PCA).**

Using PLINK 1.9, a Principal Component Analysis (PCA) was performed to identify for possible batch effects between sample data of cases and controls, which were derived from differing sources, whole-exome sequencing (WXS), and whole-genome sequencing (WGS), respectively. Figures for the were plotted in R. Focusing on the Top 2 principal components which contributed to most of the variance explained were shown in a PCA plot to showcase the clustering of samples from both data sources.

**Batch identification of gene relevance to ‘Rheumatoid Arthritis’.**

Using the Python PyMed library, a batch query interrogating the PubMed database using gene names and aliases of the selected SNPs together with the key term ‘Rheumatoid Arthritis’ was performed. Subsequently, returned results were manually curated for accuracy of gene relevancy to the disease, before being considered and evaluated within the study.

**Identifying pfSNPs in linkage disequilibrium (LD) with selected SNPs without previously established potential function.**

Using PLINK 1.9, the complete WGS data which comprises of only our control samples was used to identify all SNPs in LD with selected SNPs that were not found to have any previously established potential function at R^2^ > 0.8. The use of the complete WGS data will extend our search by allowing interrogation of SNPs that are outside of regions available from the WXS data, especially given that a majority of the selected SNPs resided outside of the coding regions. SNPs identified to be in LD were then interrogated for predicted potential function against the pfSNP database.

# **Figure S1**

**
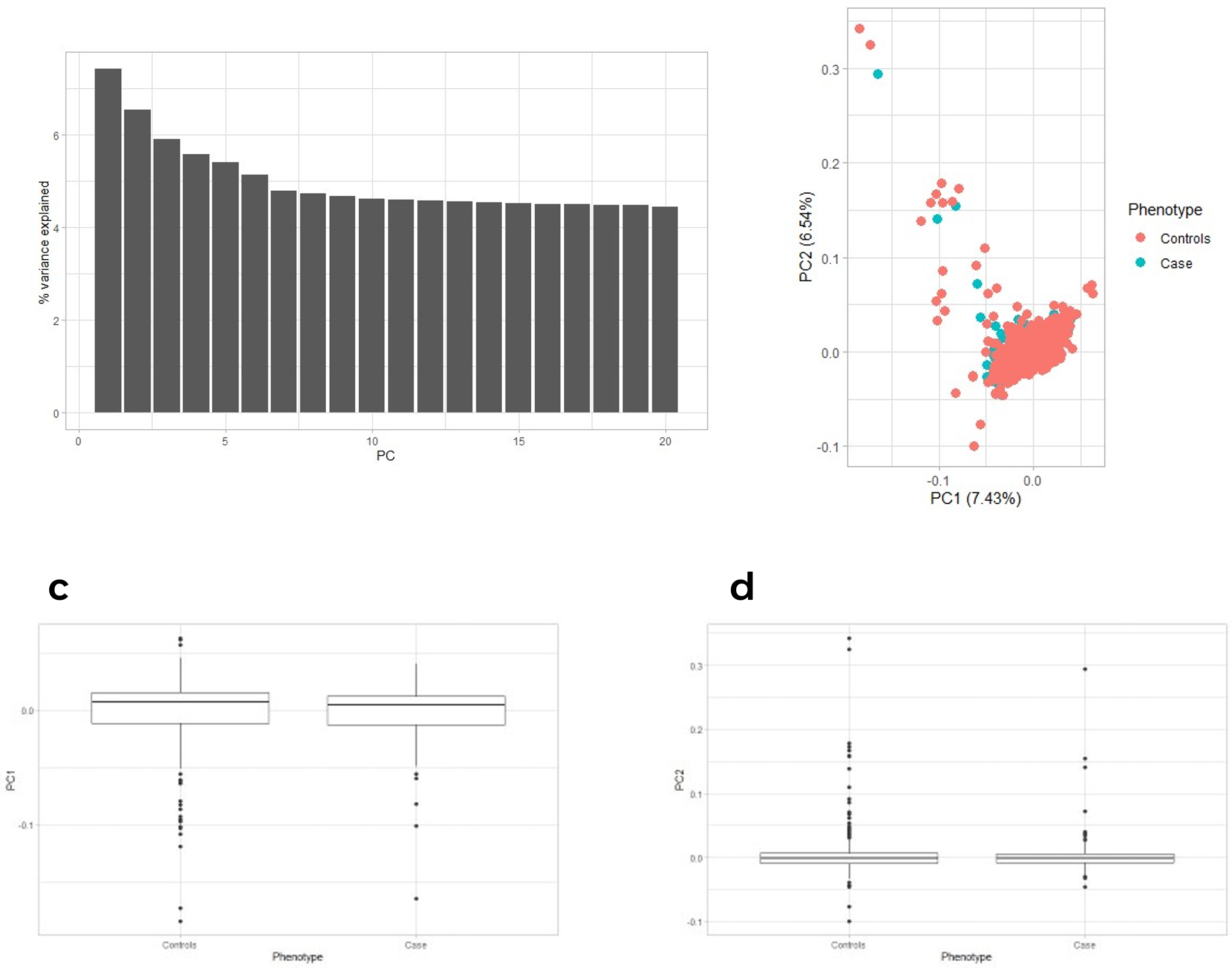
**

**Figure S1. Principal Component Analysis (PCA) of Case and Control samples from WXS and WGS respectively for the evaluation of underlying batch effects.** (a) Plot of % of variance explained by each principal component (PC). (b) PCA plot of the first two principal components of variation based on the combined case and control samples from differing data sources. (c) Boxplots of the case and control samples within PC1. (d) Boxplots of the case and control samples within PC2.

# **Figure S2**


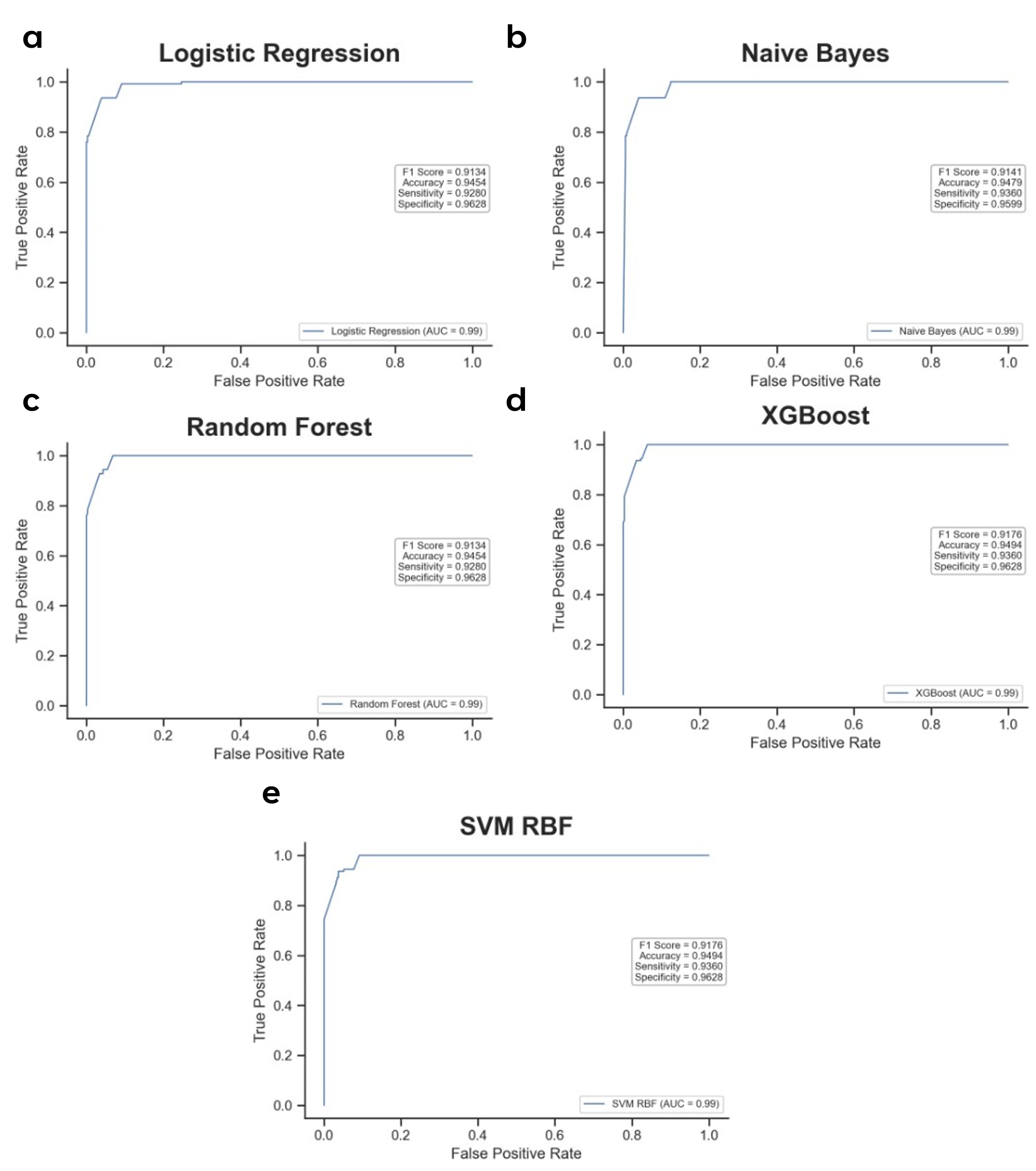


**Figure S2. Predictive performance of 9 selected SNPs in unseen Test Set 1.** ROC-AUC curves with F1 score, Accuracy, Sensitivity, and Specificity of 9 selected SNPs using (a) Logistic Regression, (b) Naïve Bayes, (c) Random Forest, (d) XGBoost, and (e) Support Vector Machine (SVM) classifiers.

# **Figure S3**


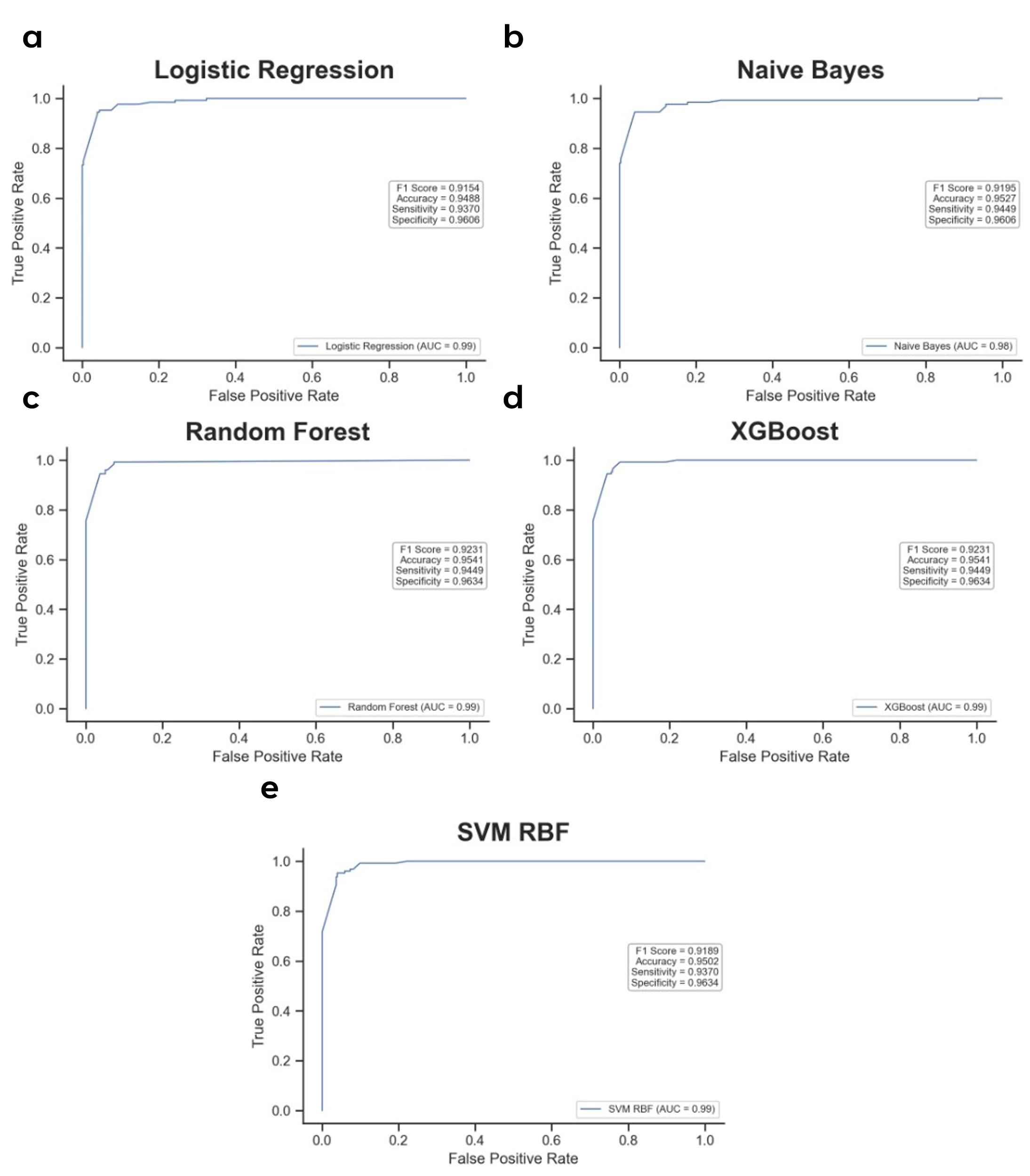


**Figure S3. Predictive performance of 9 selected SNPs in unseen Test Set 2.** ROC-AUC curves with F1 score, Accuracy, Sensitivity, and Specificity of 9 selected SNPs using (a) Logistic Regression, (b) Naïve Bayes, (c) Random Forest, (d) XGBoost, and (e) Support Vector Machine (SVM) classifiers.

# **Figure S4**


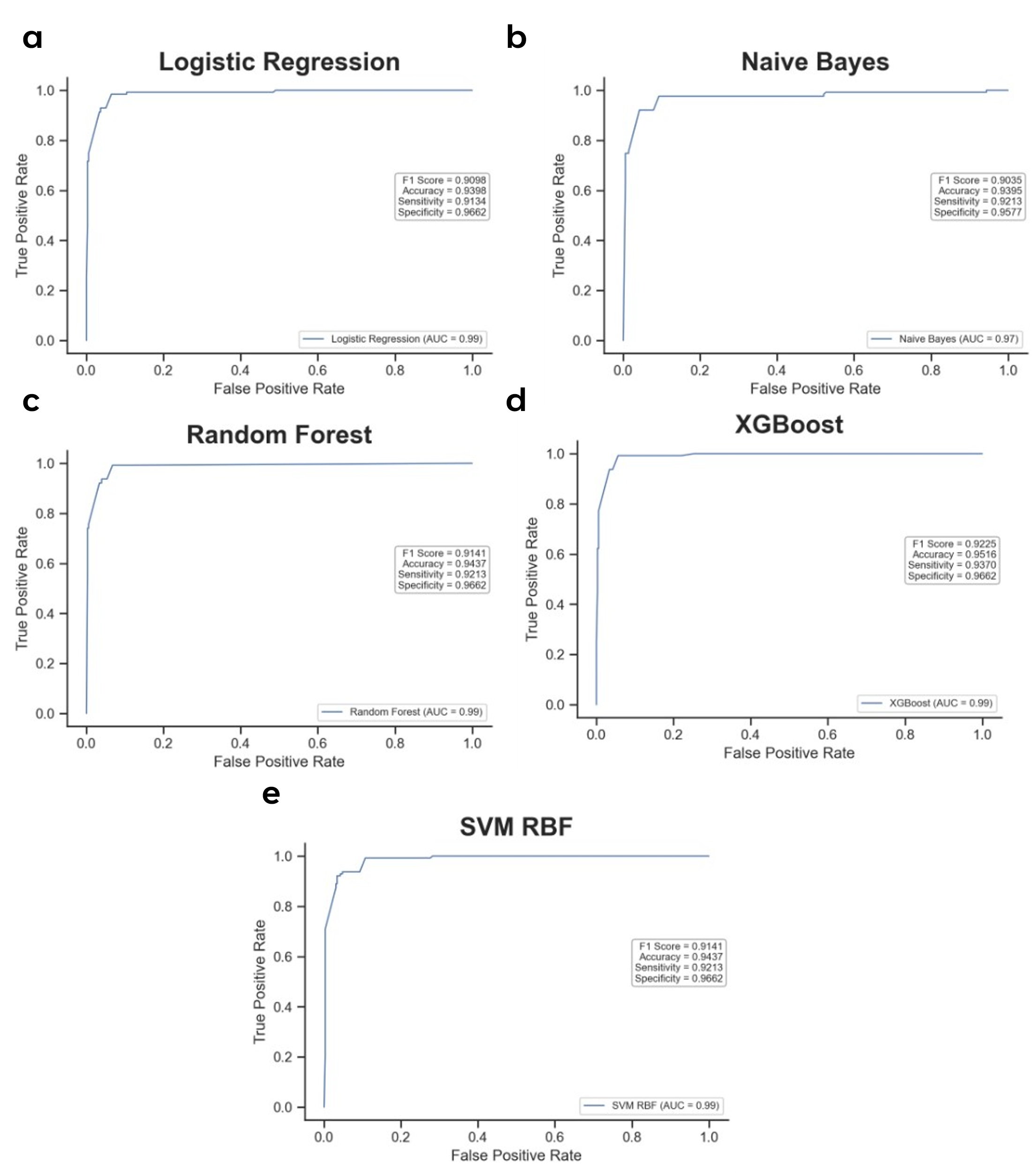


**Figure S4. Predictive performance of 9 selected SNPs in unseen Test Set 3.** ROC-AUC curves with F1 score, Accuracy, Sensitivity, and Specificity of 9 selected SNPs using (a) Logistic Regression, (b) Naïve Bayes, (c) Random Forest, (d) XGBoost, and (e) Support Vector Machine (SVM) classifiers.

# **Figure S5**


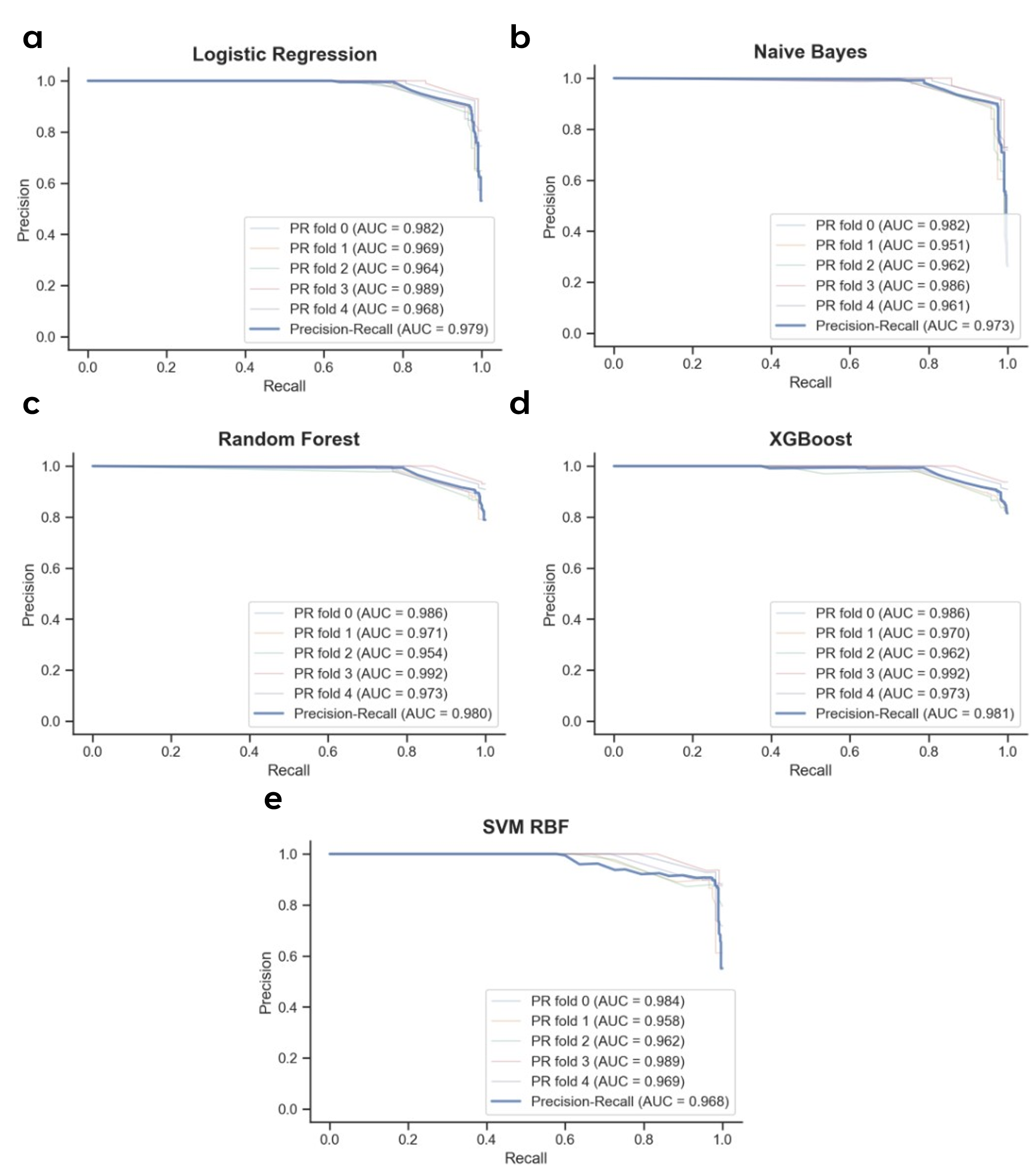


**Figure S5. Predictive performance of 9 selected SNPs in training set using 5-fold cross-validation.** Precision-Recall curves of 9 selected SNPs using (a) Logistic Regression, (b) Naïve Bayes, (c) Random Forest, (d) XGBoost, and (e) Support Vector Machine (SVM) classifiers.

# **Figure S6**


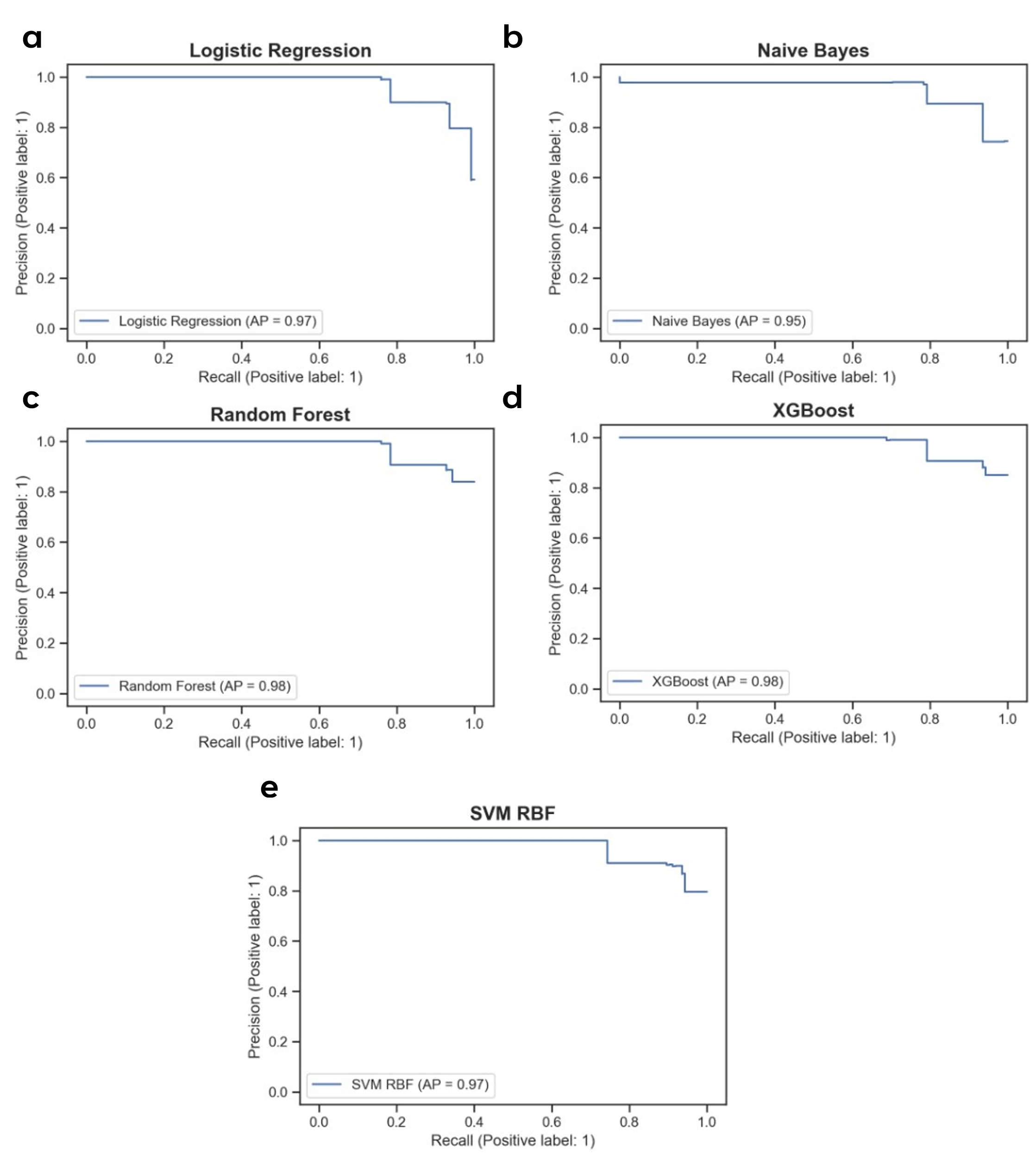


**Figure S6. Predictive performance of 9 selected SNPs in unseen Test Set 1.** Precision-Recall curves with of 9 selected SNPs using (a) Logistic Regression, (b) Naïve Bayes, (c) Random Forest, (d) XGBoost, and (e) Support Vector Machine (SVM) classifiers.

# **Figure S7**


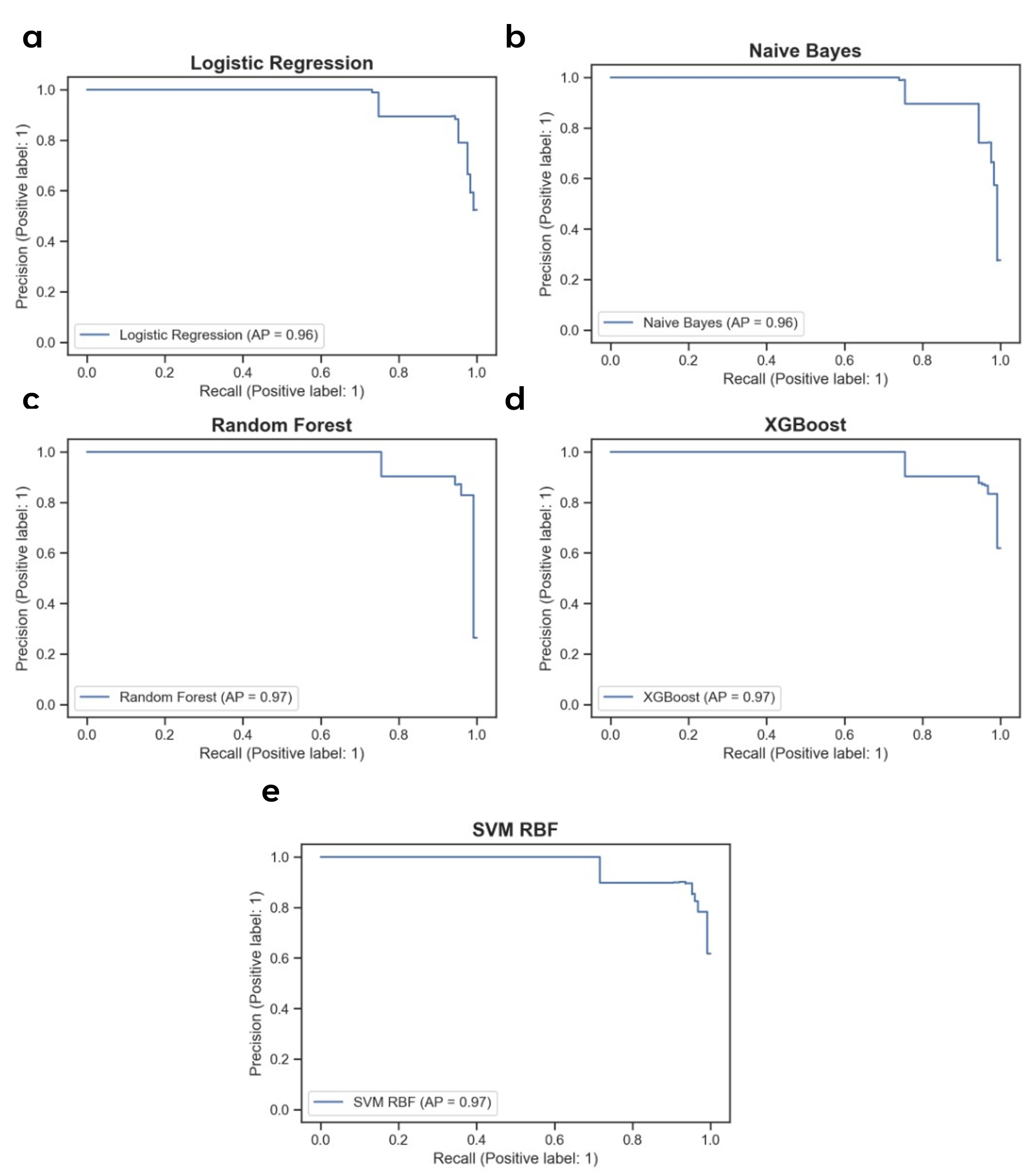


**Figure S7. Predictive performance of 9 selected SNPs in unseen Test Set 2.** Precision-Recall curves with of 9 selected SNPs using (a) Logistic Regression, (b) Naïve Bayes, (c) Random Forest, (d) XGBoost, and (e) Support Vector Machine (SVM) classifiers.

# **Figure S8**


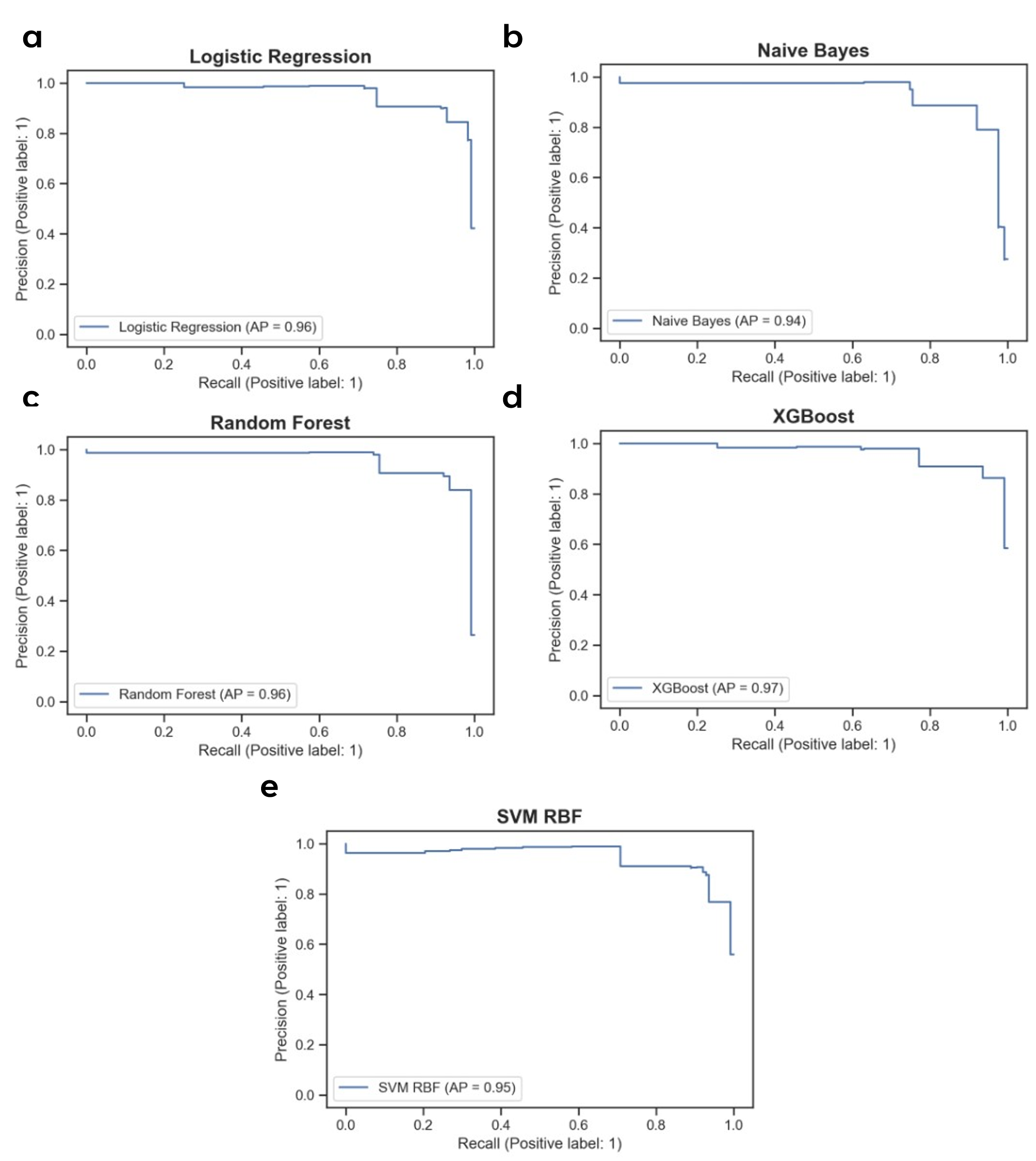


**Figure S8. Predictive performance of 9 selected SNPs in unseen Test Set 3.** Precision-Recall curves with of 9 selected SNPs using (a) Logistic Regression, (b) Naïve Bayes, (c) Random Forest, (d) XGBoost, and (e) Support Vector Machine (SVM) classifiers.

# **Figure S9**


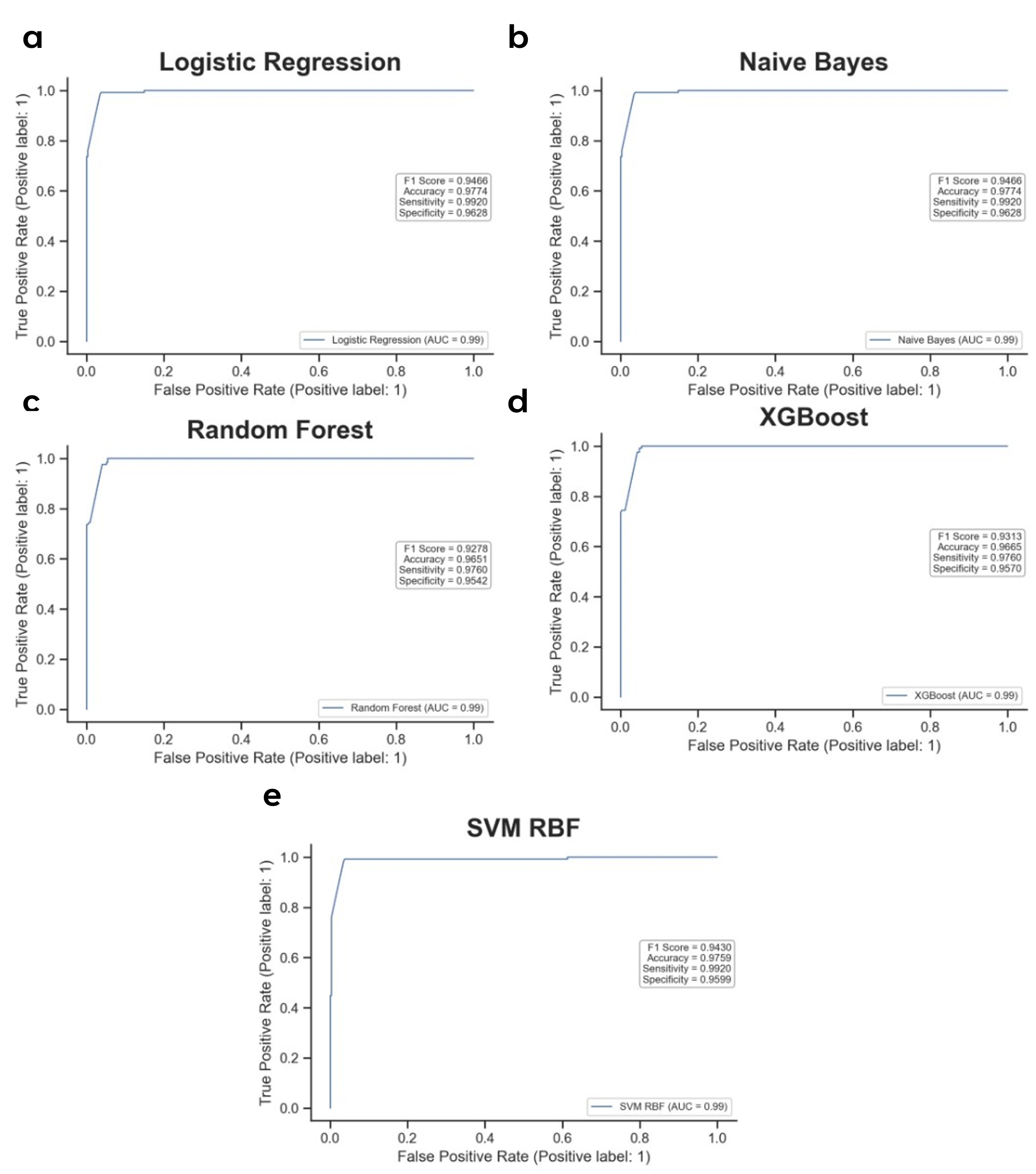


**Figure S9. Predictive performance of calculated PRS in unseen Test Set 1.** ROC-AUC curves with F1 score, Accuracy, Sensitivity, and Specificity of PRS using (a) Logistic Regression, (b) Naïve Bayes, (c) Random Forest, (d) XGBoost, and (e) Support Vector Machine (SVM) classifiers.

# **Figure S10**


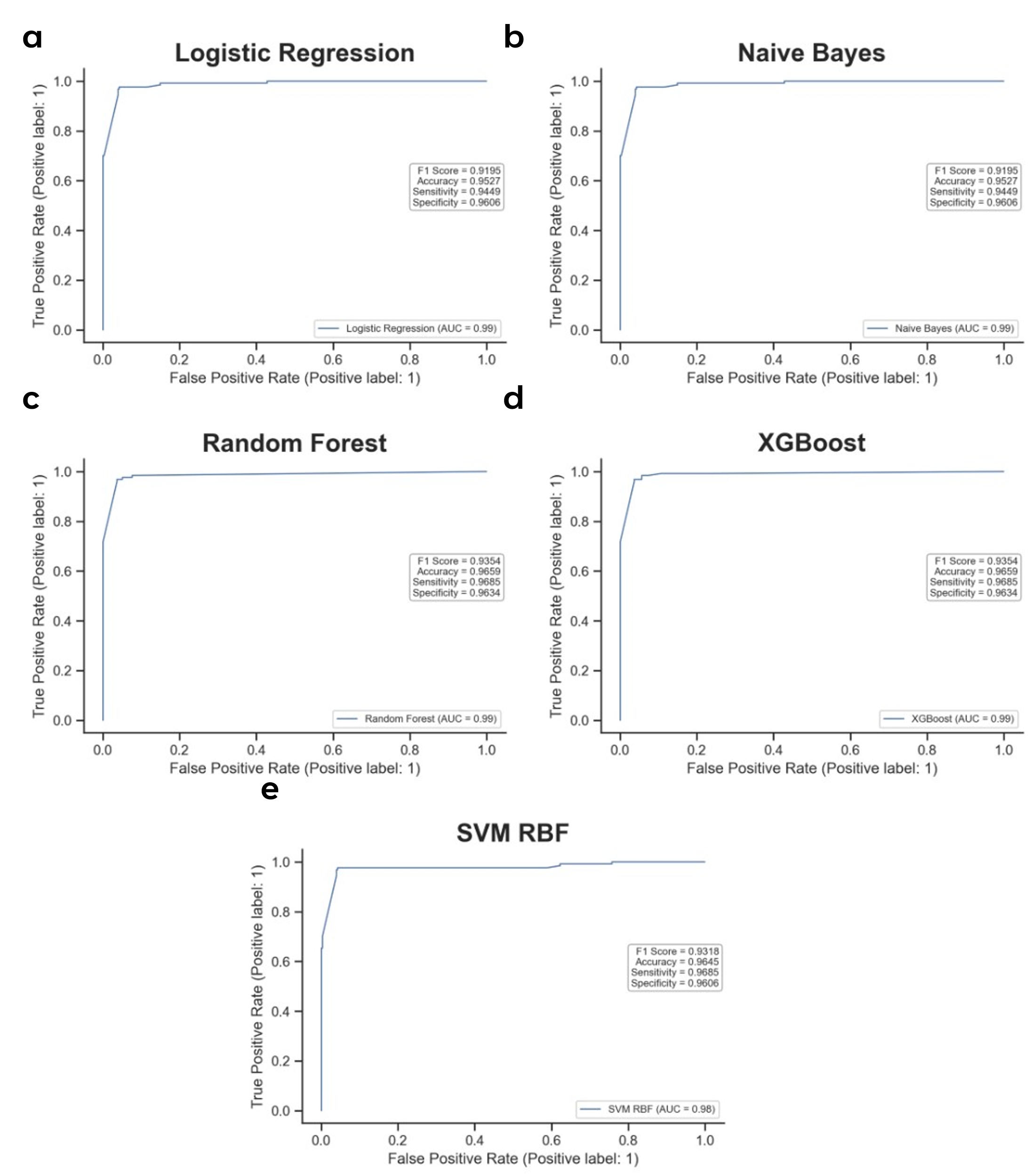


**Figure S10. Predictive performance of calculated PRS in unseen Test Set 2.** ROC-AUC curves with F1 score, Accuracy, Sensitivity, and Specificity of PRS using (a) Logistic Regression, (b) Naïve Bayes, (c) Random Forest, (d) XGBoost, and (e) Support Vector Machine (SVM) classifiers.

# **Figure S11**


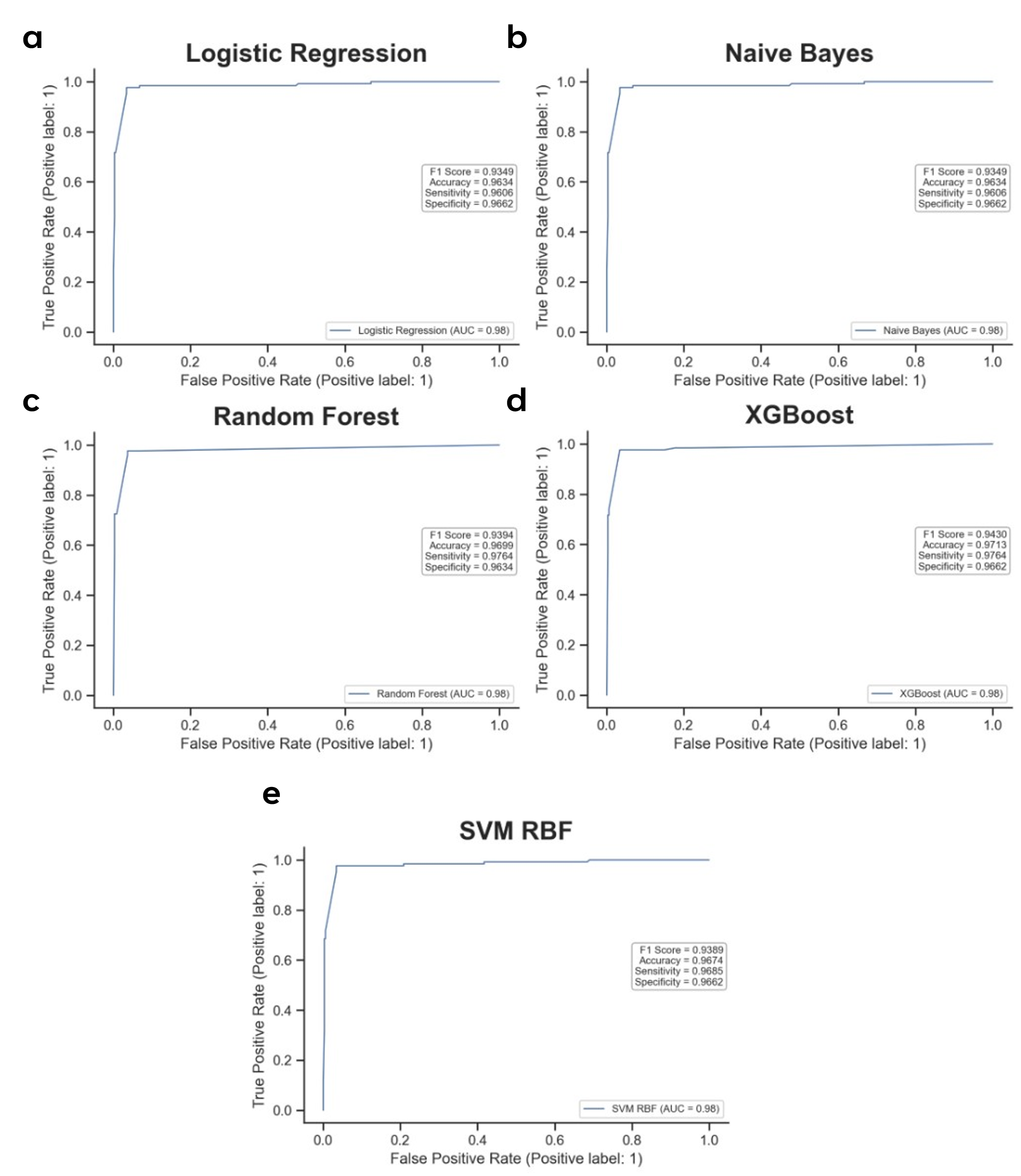


**Figure S11. Predictive performance of calculated PRS in unseen Test Set 3.** ROC-AUC curves with F1 score, Accuracy, Sensitivity, and Specificity of PRS using (a) Logistic Regression, (b) Naïve Bayes, (c) Random Forest, (d) XGBoost, and (e) Support Vector Machine (SVM) classifiers.

# **Figure S12**


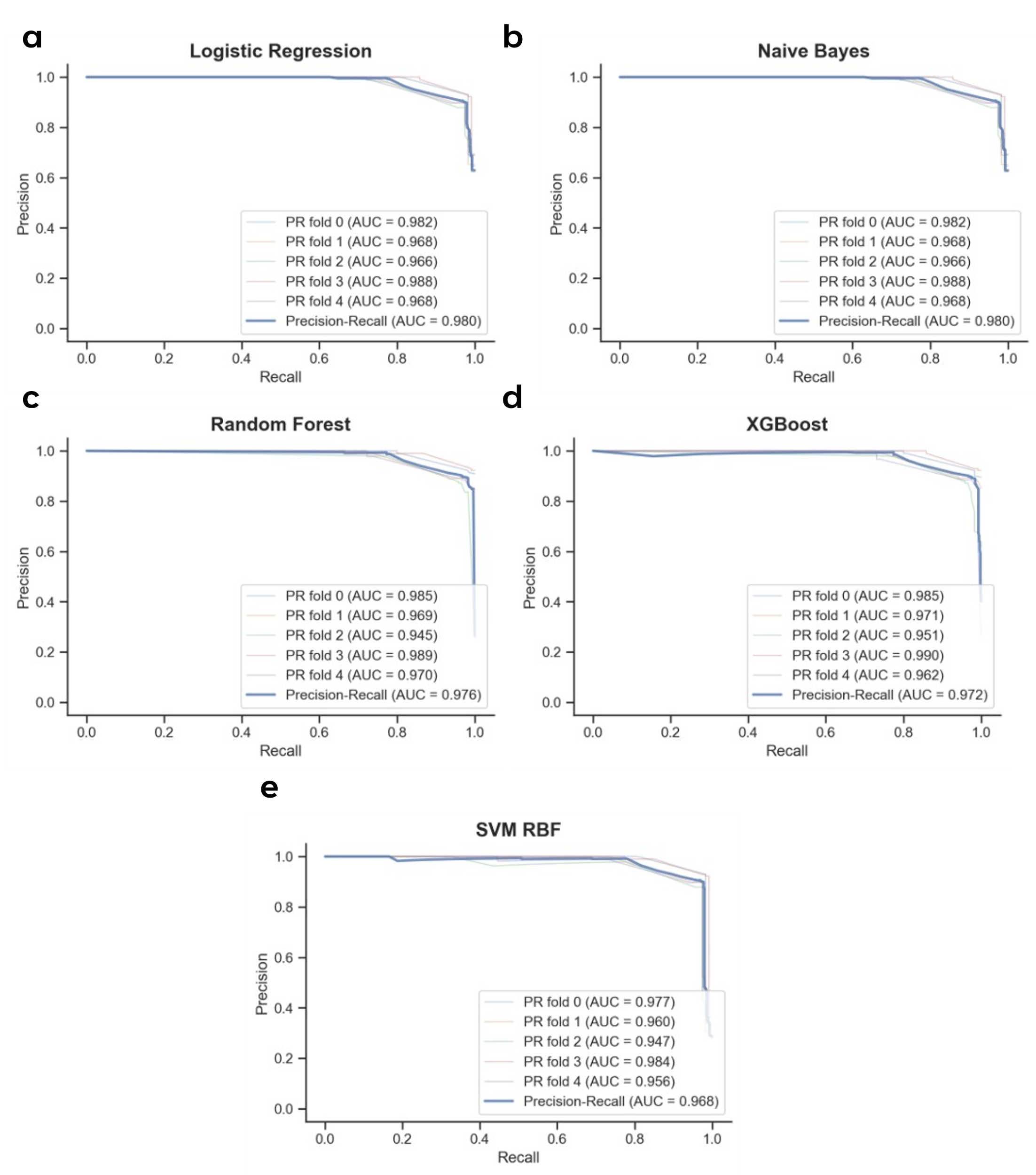


**Figure S12. Predictive performance of calculated PRS in the training set using 5-fold cross-validation.** Precision-Recall curves of PRS using (a) Logistic Regression, (b) Naïve Bayes, (c) Random Forest, (d) XGBoost, and (e) Support Vector Machine (SVM) classifiers.

# **Figure S13**


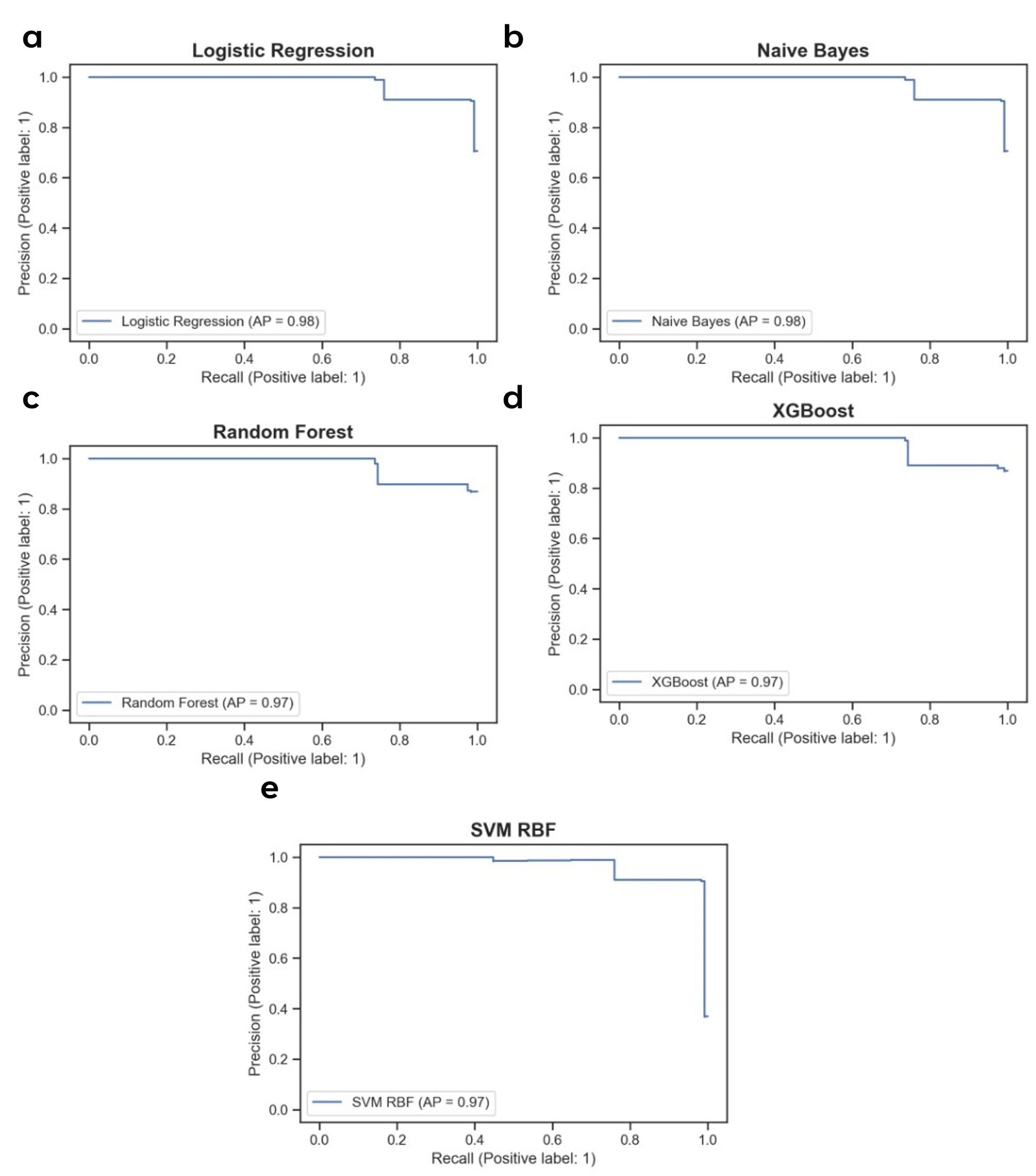


**Figure S13. Predictive performance of calculated PRS in unseen Test Set 1.** Precision-Recall curves of PRS using (a) Logistic Regression, (b) Naïve Bayes, (c) Random Forest, (d) XGBoost, and (e) Support Vector Machine (SVM) classifiers.

# **Figure S14**


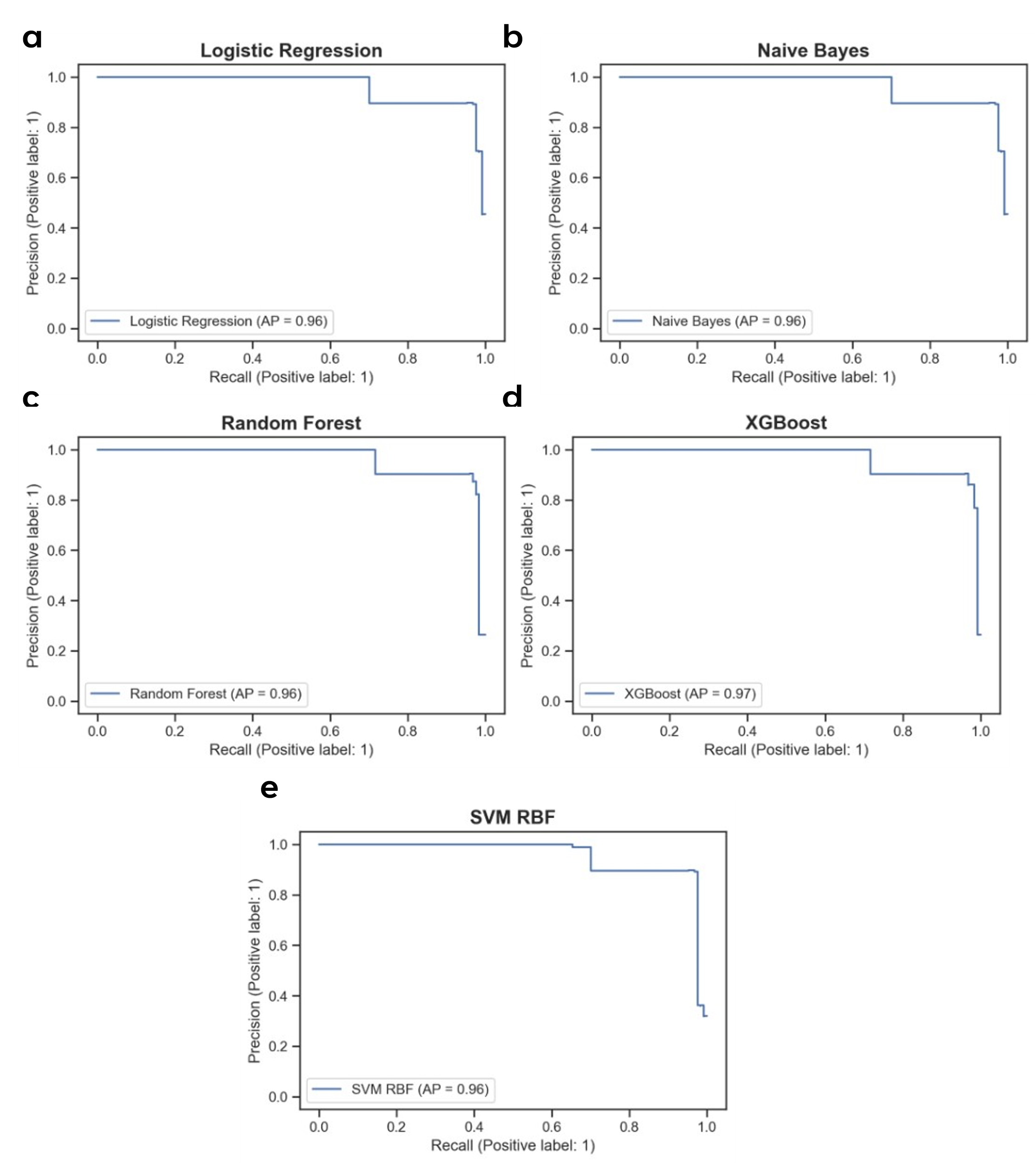


**Figure S14. Predictive performance of calculated PRS in unseen Test Set 2.** Precision-Recall curves of PRS using (a) Logistic Regression, (b) Naïve Bayes, (c) Random Forest, (d) XGBoost, and (e) Support Vector Machine (SVM) classifiers.

# **Figure S15**


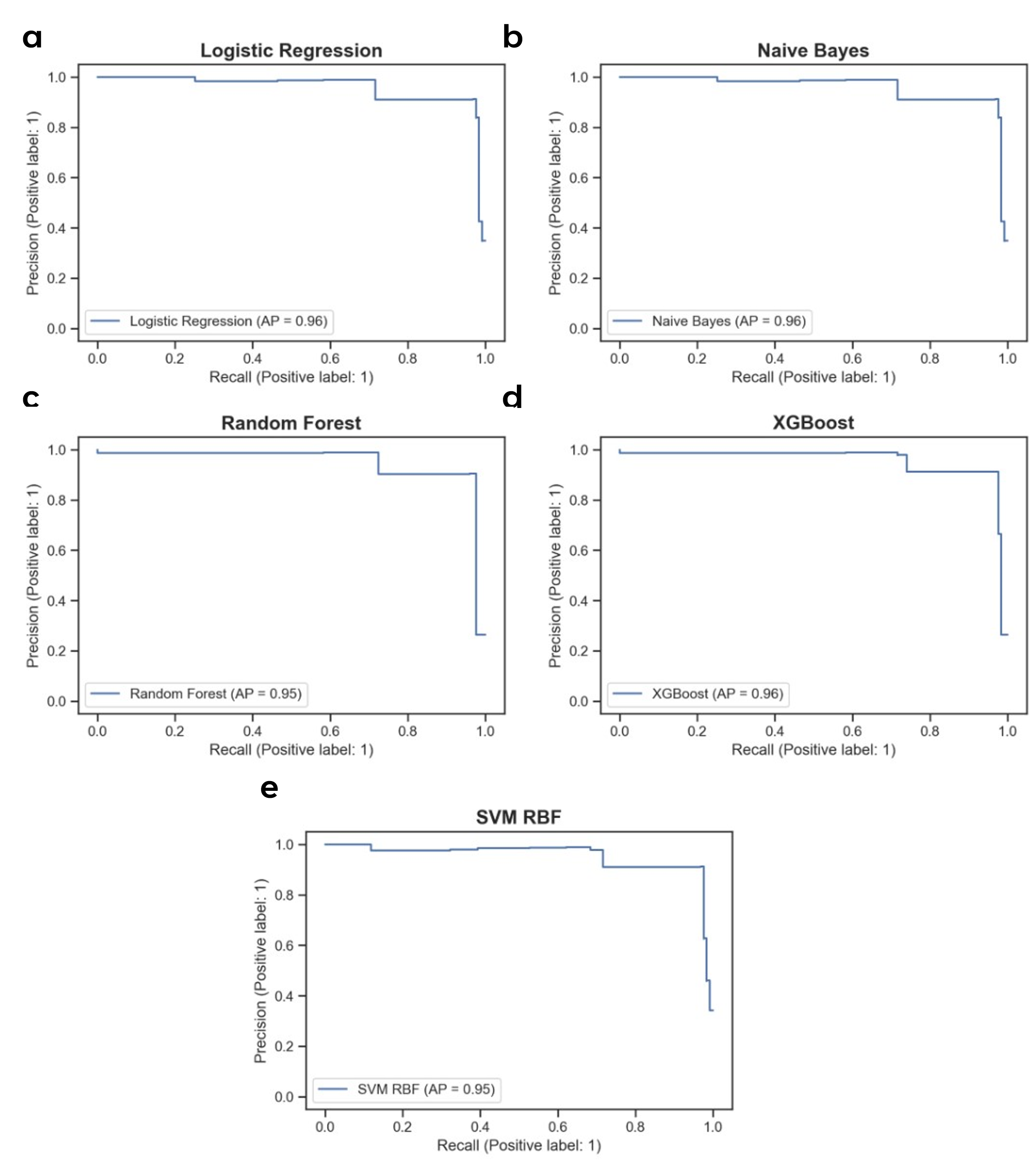


**Figure S15. Predictive performance of calculated PRS in unseen Test Set 3.** Precision-Recall curves of PRS using (a) Logistic Regression, (b) Naïve Bayes, (c) Random Forest, (d) XGBoost, and (e) Support Vector Machine (SVM) classifiers.

# **Table S1**

Summary of studies that have used of genetics in the prediction of RA.

# **Table S2**

Detailed information of the 9 selected SNPs and their genes from feature selection.

# **Table S3**

Potentially functional SNPs (pfSNPs) in linkage disequilibrium with selected SNPs without previously established potential function.

# **Table S4**

Previously identified GWAS associations of selected SNPs at p-value significance of 1 x 10^-5^ from BioBank Japan PheWeb, IEU Open GWAS Project, and GWAS Atlas.

# **Table S5**

Predictive performance of Polygenic Risk Scores (PRS) calculated from the 9 selected SNPs in a 5-fold cross-validation of the Train dataset and in each of the 3 unseen Test datasets.
