# Supplementary material for: Robust SNP-based prediction of rheumatoid arthritis through machine-learning-optimized polygenic risk score
Source: J Transl Med. 2023 Feb 7;21:92. doi: 10.1186/s12967-023-03939-5 (PMC9903430; doi:10.1186/s12967-023-03939-5)
Supplement: Supplementary file 2 — Additional file 2: Table S1–Table S5. Table S1. Summary of studies that have used of genetics in the prediction of RA. Table S2. Detailed information of the 9 selected SNPs and their genes from feature selection. Table S3. Potentially functional SNPs (pfSNPs) in linkage disequilibrium with selected SNPs without previously established potential function. Table S4. Previously identified GWAS associations of selected SNPs at p-value significance of 1 × 10^−5^ from BioBank Japan PheWeb, IEU Open GWAS Project, and GWAS Atlas. Table S5. Predictive performance of Polygenic Risk Scores (PRS) calculated from the 9 selected SNPs in a fivefold cross-validation of the Train dataset and in each of the 3 unseen Test datasets. [file 12967_2023_3939_MOESM2_ESM.pdf]

Table S1. **Summary of studies that have used of genetics in the prediction of RA**

Study	Samples	Prediction Model	Prediction Performances
**Karlson, E. W. et al.** Cumulative Association of Twenty-Two Genetic Variants with Seropositive Rheumatoid Arthritis Risk. Ann Rheum Dis 69, 1077–1085 (2010).	289 Caucasian seropositive cases and 481 controls from the US Nurses' Health Studies (NHS)<br><br>629 Caucasian CCP antibody positive cases and 623 controls from the Swedish Epidemiologic Investigation of RA (EIRA)	Weighted Genetic Risk Score (wGRS) (14 SNPs and 8 HLA alleles) with Clinical Risk Factors	**AUCs:** 0.66 - 0.75
**Kurreeman, F. et al.** Genetic Basis of Autoantibody Positive and Negative Rheumatoid Arthritis Risk in a Multi-ethnic Cohort Derived from Electronic Health Records. The American Journal of Human Genetics 88, 57–69 (2011).	1515 Electronic Health Record (EHR) derived RA cases and 1480 controls<br><br>5539 autoantibody-positive RA cases and 20169 controls from GWAS	29 RA Risk alleles (SNPs)	**AUCs:** 0.63 - 0.74
**Chibnik, L. B. et al.** Genetic Risk Score Predicting Risk of Rheumatoid Arthritis Phenotypes and Age of Symptom Onset. PLOS ONE 6, e24380 (2011).	542 Caucasian RA cases and 551 Caucasian controls from Nurses' Health Study and Nurses' Health Study II	wGRS (31 SNPs and 8 HLA alleles)	**AUCs:** 0.563 - 0.712
**Scott, I. C. et al.** Predicting the Risk of Rheumatoid Arthritis and Its Age of Onset through Modelling Genetic Risk Variants with Smoking. PLOS Genetics 9, e1003808 (2013).	Wellcome Trust Case Control Consortium (WTCCC: 1516 cases; 1647 controls);<br><br>UK RA Genetics Group Consortium (UKRAGG: 2623 cases; 1500 controls)	25 HLA alleles, 31 SNPs, Ever-smoking status	**AUCs:** 0.617 - 0.857
**Sparks, J. A. et al.** Improved performance of epidemiologic and genetic risk models for rheumatoid arthritis serologic phenotypes using family history. Annals of the Rheumatic Diseases 74, 1522–1529 (2015).	Nurses' Health Study (NHS, 381 RA cases and 410 controls)<br><br>Epidemiological Investigation of RA (EIRA, 1244 RA cases and 971 controls)	Family history, Epidemiologic Risk factors, and Genetic Risk Score (8 HLA alleles and 31 SNPs)	**AUCs:** 0.74 - 0.83
**Yarwood, A. et al.** A weighted genetic risk score using all known susceptibility variants to estimate rheumatoid arthritis risk. Annals of the Rheumatic Diseases 74, 170–176 (2015).	11366 RA cases and 15489 healthy controls from UK, USA, Sweden, The Netherlands and Spain	wGRS (45 SNPs), imputed amino acids at HLA loci, and gender	**AUC:** 0.67 - 0.78
**Rostami, S. et al.** Comparison of methods to construct a genetic risk score for prediction of rheumatoid arthritis in the population-based Nord-Trøndelag Health Study, Norway. Rheumatology 59, 1743–1751 (2020).	Nord-Trøndelag Health (HUNT) Study (489 cases; 61584 controls)	wGRS (27 SNPs)	**AUC:** 0.78
**Yu, X.-H. et al.** Systematic Evaluation of Rheumatoid Arthritis Risk by Integrating Lifestyle Factors and Genetic Risk Scores. Frontiers in Immunology 13, (2022).	UK Biobank Data (3537 cases; 278286 controls)	81 SNPs and Clinical characteristics (Age, sex, genotyped batch, Townsend deprivation index, smoking status, physical activity, drinking status, and BMI)	**AUCs:** 0.658 - 0.688

Table S2. **Detailed information of the 13 selected SNPs and their genes from feature selection**

#	SNP ID	ID	CHROM	POS	REF	ALT	Gene	Mean Feature Importance Score	Feature Importance Ranking	Univariate Logistic Regression: Odds Ratio	95% CI	β (Effect size)	P-value	Localised Region	Predicted Function (pfSNP)?	pfSNP/LD Details
1	rs1266832853	SNP1	1	248722788	G	T	OR2T29;OR2T5	0.0612	1	33.19	(23.7, 46.49)	3.502	2.57E-92	Exonic	✓	• Non-Synonymous<br>• A > D<br>• Tolerated - Low Confidence
2	rs200901373	SNP2	2	130872956	G	T	POTEF	0.0501	2	81.49	(47.68, 139.3)	4.400	3.07E-58	Intronic		
3	rs60465633	SNP3	1	16918473	G	A	NBPF1	0.0486	3	1448	(202.4, 10360)	7.278	4.20E-13	Exonic	✓	• Non-Synonymous<br>• T > M<br>• Tolerated - Low Confidence
4	rs142869031	SNP4	15	43906035	C	G	STRC	0.0456	4	0.0081	(0.0030, 0.0216)	-4.822	1.02E-21	Intronic		• 5 pfSNPs in LD
5	rs11385557	SNP5	7	97944934	A	T	BAIAP2L1	0.0365	5	0.0137	(0.0061, 0.0308)	-4.290	2.49E-25	Intronic		• 19 pfSNPs in LD
6	rs143773270	SNP6	10	18085101	G	A	TMEM236	0.0328	6	0.0053	(0.0013, 0.0213)	-5.238	1.56E-13	Exonic	✓	• Non-Synonymous<br>• G > R<br>• Deleterious
7	rs11246240	SNP7	11	651491	C	G	DEAF1	0.0268	7	0.1083	(0.0780, 0.1506)	-2.223	5.51E-40	Intronic	✓	• eQTL
8	rs112667995	SNP8	19	14675207	A	G	TECR	0.0253	8	0.0503	(0.0308, 0.0820)	-2.990	4.52E-33	Intronic	✓	• eQTL
9	rs79555231	SNP9	6	162992348	C	T	PRKN	0.0238	9	0.0825	(0.0567, 0.1200)	-2.495	7.55E-39	Intronic		• 21 pfSNPs in LD

#	GWAS Associations (10E-5): Metabolism	Immunity	Gene Expression	Disease	Endocrine System	Cellular Function	Gene Details: Full Gene Name	Gene Function	Pathways (Concised)	Pathway Source	PubMed Publications (Gene Relevance to RA)
1							Olfactory Receptor Family 2 Subfamily T Member 29; Olfactory Receptor Family 2 Subfamily T Member 5	• Odorant receptor	• Signal transduction<br>• Olfactory transduction	Reactome	
2							POTE Ankyrin Domain Family Member F	• Created from the insertion of a beta-actin fragment at the C-terminus in the POTEE paralog gene<br>• New functional chimeric protein.			
3							NBPF Member 1	• Encoded by one of the numerous copies of NBPF genes clustered in the p36, p12 and q21 region of the chromosome 1.			
4							Stereocilin	• Essential to the formation of horizontal top connectors between outer hair cell stereocilia	• Sensory Perception	Reactome	
5	✓		✓	✓	✓		BAR/IMD Domain Containing Adaptor Protein 2 Like 1	• May function as adapter protein<br>• Involved in the formation of clusters of actin bundles • Plays a role in the reorganization of the actin cytoskeleton in response to bacterial infection	• Signal transduction	Reactome	**Distinctive gene expression signatures in rheumatoid arthritis synovial tissue fibroblast cells: correlates with disease activity.** Genes Immun. 2007 86 8, 480–491 (2007).<br><br>Galligan, C. L., Baig, E., Bykerk, V., Keystone, E. C. & Fish, E. N.
6							Transmembrane Protein 236	• Predicted to be integral component of membrane			
7			✓				DEAF1 Transcription Factor	• Transcription factor that binds to sequence with multiple copies of 5'-TTC[CG]G-3' present in its own promoter and that of the HNRPA2B1 gene.<br>• Down-regulates transcription of these genes.<br>• Activates the proenkephalin gene independently of promoter binding<br>• Inhibitor of cell proliferation, by arresting cells in the G0 or G1 phase.<br>• Required for neural tube closure and skeletal patterning.	• SIDS Susceptiblity Pathways	Wikipathways	
8	✓		✓			✓	Trans-2,3-Enoyl-CoA Reductase	Production of very long-chain fatty acids for sphingolipid synthesis<br>• Degradation of the sphingosine moiety in sphingolipids<br>• Catalyzes the last of the four reactions of the long-chain fatty acids elongation cycle	• Metabolism of Lipids	Reactome	
9							Parkin RBR E3 Ubiquitin Protein Ligase	• Promotes the autophagic degradation of dysfunctional depolarized mitochondria via the promotion of ubiquitination of mitochondrial proteins	• Metabolism of Proteins<br>• Adaptive Immune System<br>• Mitophagy	Reactome	**Loss of Parkin reduces inflammatory arthritis by inhibiting p53 degradation.** Redox Biol. 12, 666 (2017).<br><br>Jung, Y. Y. et al.

Table S3. Potentially functional SNPs (pfSNPs) in linkage disequilibrium with selected SNPs without previously established potential function.

SNP	LD SNPs	Gene	Localised Region	Coding Region Details	Predicted Function	pfSNP Details	R2
rs142869031	rs2614819	STRC	Intronic		E		0.935
	rs2614822	STRC	Intronic		E		0.935
	rs2729509	STRC	Exonic	• F>C | F>S<br>• Non-Synonymous	S	• FB_ESE_222 (+)	0.814
	rs3101443	STRC	Intergenic		T/E	• cap (-) | GEN_INI (-) | AREB6 (+)	0.952
	rs2614811	STRC	Intergenic		T/E	• Nkx2-5 (+) | Opaque-2 (+) | Tst-1 (+)	0.952
rs11385557	rs13232181	BAIAP2L1	Intronic		E		0.997
	rs12673586	BAIAP2L1	Intronic		E		0.994
	rs6465666	BAIAP2L1	Intronic		E		0.994
	rs2394852	BAIAP2L1	Intronic		E		1.000
	rs7791321	BAIAP2L1	Intronic		E		0.997
	rs2107716	BAIAP2L1	Intronic		I/E	• GY_DS_ISRE_78 (+) | GY_DS_ISRE_138 (-) | GY_DS_ISRE_145 (-)	1.000
	rs10647784	BAIAP2L1	Intronic		I	• GY_DS_ISRE_58 (-)	0.986
	rs6963210	BAIAP2L1	Intronic		I/E	• GY_DS_ISRE_20 (-) | GY_DS_ISRE_50 (-) | GY_DS_ISRE_89 (-)	1.000
	rs9648908	BAIAP2L1	Intronic		I/E	• GY_DS_ISRE_68 (+) | GY_DS_ISRE_130 (-)	1.000
	rs7797943	BAIAP2L1	Intronic		I/E	• GY_DS_ISRE_73 (-)	1.000
	rs3801264	BAIAP2L1	Intronic		E		1.000
	rs1468338	BAIAP2L1	Intronic		E		1.000
	rs2158553	BAIAP2L1	Intronic		E		1.000
	rs12112351	BAIAP2L1	Intronic		E		1.000
	rs10808107	BAIAP2L1	Intronic		E		1.000
	rs10953256	BAIAP2L1	Intronic		E		1.000
	rs2286074	BAIAP2L1	Intronic		E		1.000
	rs7458551	BAIAP2L1	Intronic		E		1.000
rs79555231	rs10080731	PRKN	Intronic		T	• En-1 (-) | Msx-1 (-)	1.000
	rs10080795	PRKN	Intronic		T	• MEIS1B:HOXA9 (-) | Ubx (-)	1.000
	rs10080853	PRKN	Intronic		T	• GR (-) | MCM1+SFF (-) | AG (+)	1.000
	rs11966654	PRKN	Intronic		T	• HMGIY (-) | Oct-4 (POU5F1) (-) | AIRE (+) | c-Rel (+)	1.000
	rs11967554	PRKN	Intronic		T	• MATalpha2 (+) | TGA1a (+)	1.000
	rs11968095	PRKN	Intronic		T	• Opaque-2 (+) | Pax-6 (+)	1.000
	rs11968987	PRKN	Intronic		T	• OCT1 (-) | Pbx-1 (-) | HOXA4 (+)	0.995
	rs11969575	PRKN	Intronic		T	• C/EBPgamma (-)	0.998
	rs35988208	PRKN	Intronic		T	• GATA-2 (-)	1.000
	rs4091546	PRKN	Intronic		T	• AREB6 (-) | HSF (-)	1.000
	rs6906849	PRKN	Intronic		T	• GR (-)	1.000
	rs7450548	PRKN	Intronic		T	• cap (-) | IRF (-)	0.911
	rs7451147	PRKN	Intronic		T	• HSF (+) | Oct-4 (POU5F1) (+)	1.000
	rs6929604	PRKN	Intronic		P/I	• LIM1 (-)<br>• GY_DS_ISRE_68 (+) | GY_DS_ISRE_130 (-)	1.000
	rs9458598	PRKN	Intronic		P	• AML1 (-) | AML1a (-) | core-binding factor (-)	0.907
	rs9458599	PRKN	Intronic		P	• FACB (-) | Ncx (+) | Tst-1 (+)	1.000
	rs9458600	PRKN	Intronic		P	• Gfi1b (-)	0.911
	rs9458601	PRKN	Intronic		P	• Crx (+)	0.911
	rs9458602	PRKN	Intronic		P	• OCT1 (-) | POU3F2 (-) | v-Myb (+)	1.000
	rs9458603	PRKN	Intronic		P	• MADS-A (-) | YY1 (-) | BR-C Z2 (+)	1.000
	rs6934979	PRKN	Intronic		I	• GY_DS_ISRE_40 (+) | GY_DS_ISRE_5 (-)	1.000

pf-Legend:	
T	Alter TF/miRNA binding site
I	ISRE
P	Alter Promoter site
E	Alter Gene Expression (eQTL)
S	Exonic Splice Enhancer/Silencer (ESE/ESS)

<u>Table S4.</u> Previously identified GWAS associations of selected SNPs at p-value significance of 1 x 10-5 from BioBank Japan PheWeb, IEU Open GWAS Project, and GWAS Atlas.

#	Database	Assoc. Category	Associations	rs11385557	rs11246240	rs112667995
			SNP rsID	rs11385557	rs11246240	rs112667995
			Gene	BAIAP2L1	DEAF1	TECR
#	Database	Assoc. Category	Associations	p-values		
1	OpenGWAS	Immunity	Lymphocyte count	1.94E-05		
2	OpenGWAS		Lymphocyte percentage	6.39E-12		
3	OpenGWAS		Lymphocyte percentage of white cells	9.08E-05		
4	OpenGWAS		Monocyte count	1.78E-05		
5	OpenGWAS		Neutrophill count	5.32E-05		
6	OpenGWAS		Neutrophill percentage	1.11E-08		
7	OpenGWAS	Diseases	L72 Follicular cysts of skin and subcutaneous tissue	4.91E-05		
8	OpenGWAS		Other peripheral nerve disorders	7.69E-05		
9	OpenGWAS		Prostate cancer	1.27E-11		
10	OpenGWAS	Endocrine System	SHBG	3.69E-26		
11	OpenGWAS		Testosterone	6.81E-09		
12	OpenGWAS	Cell Function	Kinesin-like protein KIF23			7.41E-05
13	Biobank Japan PheWeb	Metabolism	Alkaline phosphatase	6.14E-07		
14	OpenGWAS		Low density lipoprotein cholesterol levels	1.20E-05		
15	OpenGWAS		Cystatin C			8.47E-06
16	OpenGWAS		Urate	2.48E-06		
17	OpenGWAS	Gene Expression	eQTL-ANO9		1.56E-07	
18	OpenGWAS		eQTL-ASNS	4.63E-11		
19	OpenGWAS		eQTL-B4GALNT4		3.94E-05	
20	OpenGWAS		eQTL-BRI3	2.87E-11		
21	OpenGWAS		eQTL-CLEC17A			1.01E-05
22	OpenGWAS		eQTL-DEAF1		5.93E-11	
23	OpenGWAS		eQTL-DNAJB1			9.47E-07
24	OpenGWAS		eQTL-DRD4		1.99E-14	
25	OpenGWAS		eQTL-EPS8L2		2.85E-06	
26	OpenGWAS		eQTL-GIPC1			4.23E-05
27	OpenGWAS		eQTL-HRAS		2.01E-08	
28	OpenGWAS		eQTL-LRRC56		6.35E-36	
29	OpenGWAS		eQTL-PHRF1		3.98E-06	
30	OpenGWAS		eQTL-PIDD1		1.10E-08	
31	OpenGWAS		eQTL-RNH1		7.01E-10	
32	OpenGWAS		eQTL-TECR			2.70E-05

Table S5. **Predictive performance of Polygenic Risk Scores (PRS) calculated from the 13 selected SNPs in a 5-fold cross-validation of the Training dataset and in each of the 3 unseen Test datasets.**

		Machine Learning Models				
**Dataset**	**Evaluation Metric**	**Logistic Regression**	**Naïve Bayes**	**Random Forest**	**XGBoost**	**SVM RBF**
**Training set Cross-validation**	**Mean AUC**	0.992	0.992	0.992	0.992	0.982
	**Mean Sensitivity**	0.967	0.965	0.970	0.972	0.972
	**Mean Specificity**	0.964	0.964	0.961	0.960	0.962
	**Mean Accuracy**	0.965	0.965	0.966	0.966	0.967
	**Mean Average Precision (PR-AUC)**	0.980	0.980	0.976	0.972	0.968

		Machine Learning Models				
**Dataset**	**Evaluation Metric**	**Logistic Regression**	**Naïve Bayes**	**Random Forest**	**XGBoost**	**SVM RBF**
**Test1 Evaluation**	**AUC**	0.994	0.994	0.993	0.992	0.990
	**Sensitivity**	0.992	0.992	0.976	0.976	0.992
	**Specificity**	0.963	0.963	0.954	0.957	0.960
	**F1 Score**	0.947	0.947	0.928	0.931	0.943
	**Accuracy**	0.977	0.977	0.965	0.967	0.976
	**Avg. Precision**	0.977	0.977	0.973	0.972	0.970
**Test2 Evaluation**	**AUC**	0.988	0.988	0.986	0.989	0.978
	**Sensitivity**	0.945	0.945	0.969	0.969	0.969
	**Specificity**	0.961	0.961	0.963	0.963	0.961
	**F1 Score**	0.920	0.920	0.935	0.935	0.932
	**Accuracy**	0.953	0.953	0.966	0.966	0.965
	**Avg. Precision**	0.962	0.962	0.962	0.966	0.955
**Test3 Evaluation**	**AUC**	0.984	0.984	0.980	0.983	0.983
	**Sensitivity**	0.961	0.961	0.976	0.976	0.969
	**Specificity**	0.966	0.966	0.963	0.966	0.966
	**F1 Score**	0.935	0.935	0.939	0.943	0.939
	**Accuracy**	0.963	0.963	0.970	0.971	0.967
	**Avg. Precision**	0.959	0.959	0.949	0.955	0.953
